# Deep insights into the response of human cervical carcinoma cells to a new cyano enone-bearing triterpenoid soloxolone methyl: a transcriptome analysis

**DOI:** 10.18632/oncotarget.27085

**Published:** 2019-09-03

**Authors:** Andrey V. Markov, Alexander E. Kel, Oksana V. Salomatina, Nariman F. Salakhutdinov, Marina A. Zenkova, Evgeniya B. Logashenko

**Affiliations:** ^1^ Institute of Chemical Biology and Fundamental Medicine, Siberian Branch of the Russian Academy of Sciences, Novosibirsk 630090, Russian Federation; ^2^ geneXplain GmbH, Wolfenbüttel 38302, Germany; ^3^ N. N. Vorozhtsov Novosibirsk Institute of Organic Chemistry, Siberian Branch of the Russian Academy of Sciences, Novosibirsk 630090, Russian Federation

**Keywords:** soloxolone methyl, cervical carcinoma, CDDO-Me, cytoscape, mechanism of action

## Abstract

Semisynthetic triterpenoids, bearing cyano enone functionality in ring A, are considered now as novel promising anti-tumor agents. However, despite the large-scale studies, their effects on cervical carcinoma cells and, moreover, mechanisms underlying cell death activation by such compounds in this cell type have not been fully elucidated. In this work, we attempted to reconstitute the key pathways and master regulators involved in the response of human cervical carcinoma KB-3-1 cells to the novel glycyrrhetinic acid derivative soloxolone methyl (SM) by a transcriptomic approach. Functional annotation of differentially expressed genes, analysis of their *cis^-^*regulatory sequences and protein-protein interaction network clearly indicated that stress of endoplasmic reticulum (ER) is the central event triggered by SM in the cells. A range of key ER stress sensors and transcription factor AP-1 were identified as upstream transcriptional regulators, controlling the response of the cells to SM. Additionally, by using Gene Expression Omnibus data, we showed the ability of SM to modulate the expression of key genes involved in regulation of the high proliferative rate of cervical carcinoma cells. Further Connectivity Map analysis revealed similarity of SM's effects with known ER stress inducers thapsigargin and geldanamycin, targeting SERCA and Grp94, respectively. According to the molecular docking study, SM could snugly fit into the active sites of these proteins in the positions very close to that of both inhibitors. Taken together, our findings provide a basis for the better understanding of the intracellular processes in tumor cells switched on in response to cyano enone-bearing triterpenoids.

## INTRODUCTION

Cervical cancer (CC) remains one of the leading causes of cancer-related deaths around the world. This disease ranks as fourth most frequently diagnosed cancer and the fourth leading cause of cancer death in women with an estimated 570,000 cases and 311,000 deaths in 2018 worldwide [1]. Nowadays, a decline in the incidence of CC in developed countries is observed, however, in developing countries this index is continuing to increase [1]. This fact has been linked to the lack of proper screening, early diagnosis and ineffective treatment protocols [2]. In spite of development of CC-targeted drugs, like erlotinib, the efficiency of current therapies is limited due to cancer clonal and intratumor heterogeneities and cell signaling complexity [6]. Therefore, the search for and development of novel effective therapeutic drugs for cancer treatment are highly important tasks.

**Figure 1:**
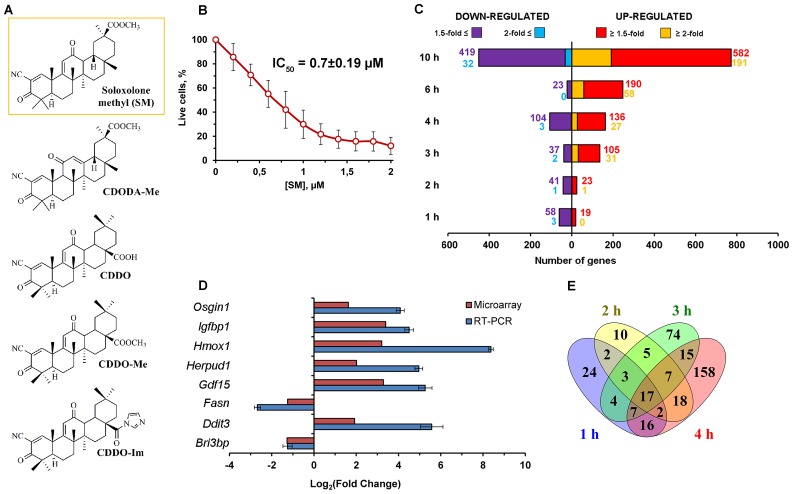
Effect of SM on transcriptome of KB-3-1 human cervical carcinoma cells. (**A**) Chemical structures of cyano enone-bearing semisynthetic triterpenoids. The structure of the investigated derivative SM was marked by the orange line. (**B**) The effect of SM on viability of KB-3-1 cells. The cells were treated by indicated concentrations of SM for 24 h and then cell viability was measured by MTT assay. Error bars represent the standard deviation of six independent experiments performed in tri- or tetraplicate. (**C**) The number of DEGs (*p* < *0.05*) between KB-3-1 cells treated by 1 μM of SM and untreated samples for the mentioned time intervals. (**D**) RT-PCR validation of the microarray results. Results of RT-PCR validation were from three independent experiments and were normalized to *GAPDH*. Relative expression levels were shown as mean ± standard deviation. (**E**) Venn diagram illustrating the overlap between DEGs identified in KB-3-1 cells during the early period of SM treatment.

Natural metabolites play an important role in antitumor drug development. These compounds are characterized by a wide spectrum of bioactivities, high diversity in chemical structures, lesser toxicity in comparison with products of chemical synthesis and the ability to effect on multiple targets in an organism, displaying additive and synergic pharmacological effects [3]. Pentacyclic triterpenoids (PTs) are one of the most extensively investigated classes of natural compounds. In a wide range of works it was shown that PTs display antiproliferative activity against a huge range of tumor cells, causing cell cycle arrest, the triggering of apoptosis and autophagy and inhibiting the growth and metastasis of tumors on different murine transplantable, xenograft and carcinogen-induced tumor models *in vivo* [4]. In recent decades, increasing research efforts were devoted to the development of chemical derivatives of PTs, with the goal of obtaining more pronounced biological activity and selectivity of action. Some of the most successful examples of semisynthetic triterpenoids are the oleanolic acid derivative CDDO-Me and its analogs (Figure 1A), bearing cyano enone functionality in ring A [4]. Such compounds were shown to be not only cytotoxic for tumor cells, but also can modify tumor microenvironment by inducing phase 2 detoxifying enzymes’ expression [5], inhibiting inflammation response [5, 6] and triggering repolarization of tumor associated macrophages to M1 phenotype [7], thus displaying a complex effect on tumor growth. Now, CDDO-Me and its fluorine-containing analogue RTA408 have currently reached the clinical trial stage for the treatment of advanced solid tumors and lymphoid malignancies [8], as well as non-small cell lung carcinoma and melanoma [9, 10]. Examples of other CDDO-Me related triterpenoids actively investigated nowadays are cyano enone-containing derivatives of glycyrrhetinic acid soloxolone methyl (SM), also known as CDODO-Me-12 [6, 11–13], and CDODA-Me [14].

Analysis of published data, performed by us, revealed poor understanding of mechanisms underlying the antitumor effect of PTs in CC cells (Supplementary Table 1). This fact can be explained by the multitarget mode of action of PTs in tumor cells and the high complexity of cellular signaling [4]. In order to deeply understand the intracellular perturbations caused by triterpenoids and reveal their primary targets, a complex view of the processes induced by the compounds in cells is highly needed. This task can be accomplished by using omics techniques, providing a powerful tool for the comprehensive analysis of cells’ responses to compound treatment in transcriptomic, proteomic or metabolic levels and providing an opportunity to reconstruct the mechanism of its action [15].

In this study, we analyzed the responses of human cervical carcinoma KB-3-1 cells to treatment with cyano enone-bearing semisynthetic triterpenoid SM (Figure 1A, compound in the orange frame) at the transcriptomic level. Previously, our screening of cytotoxicity of SM on a panel of tumor cells revealed high sensitivity of KB-3-1 cells to SM treatment [11]. Subsequently we showed that SM induces G_2_/M arrest and mitochondrial- and caspase-dependent apoptosis in this cell line [11]. Here, we tried to reveal the key processes, induced by the triterpenoid in KB-3-1 cells, that preceded the triggering of apoptosis, and probable master regulators of such perturbations by using a cDNA microarray approach. We found that SM displays marked endoplasmic reticulum (ER)-targeted action and triggers cytoprotective compensatory mechanisms in the cells, which, however, does not fully eliminate SM-induced stress. We also revealed that SERCA and GRP94 can be considered as probable primary targets of SM, the inhibiting of which by triterpenoids leads to the triggering ER stress in tumor cells. These results show a novel possible mechanism of action of cyano enone-bearing triterpenoids and provide a basis for further target-guided optimization of the structure of triterpenoid derivatives as potential anticancer therapeutics.

## RESULTS

### Microarray analysis of SM-treated human cervical carcinoma cells

In order to analyze the effect of SM on human cervical carcinoma KB-3-1 cells and identify its probable primary targets and mechanism of antiproliferative action a microarray approach was performed.

On the first step of the study we evaluated cytotoxicity of SM in the model cells and revealed that triterpenoid effectively inhibits their viability at low micromolar level (IC_50_ = 0.70 ± 0.19 μM) (Figure 1B). The selection of a correct working concentration of SM for microarray analysis was based on the fact that cyano enone-bearing triterpenoids can display different biological responses depending on used doses – according to Liby and Sporn, nanomolar concentrations of these compounds show cytoprotective and anti-inflammatory effects, whereas their higher concentrations (micromolar) can selectively induce apoptosis in cancer cells [16]. As we focus our attention on the mechanisms underlying precisely the pro-apoptogenic activity of SM, we decided to use 1 μM of the compound as the working concentration. Moreover, according to our recent review [4], similar concentrations were used in the majority of works investigated the mechanisms of antitumor effects of other cyano enone-bearing triterpenoids that can give opportunity to compare our results with these data. Previously we showed that SM at 1 μM induced apoptotic changes in KB-3-1 cells within 18 h of the treatment [11].

Further, the SM’s effect on transcriptome of KB-3-1 cells was analyzed by using Illumina Human HT-12 v4 Expression BeadChips during the first 10 h of treatment because this time interval covered both early cellular response to triterpenoid (1–4 h) and the late phase of its action (6–10 h), when the main events leading to the activation of programmed cell death were thought to occur. After data normalization, we compared gene expression in SM-treated cells with those in untreated cells and computed fold changes. The diagram depicted in Figure 1C shows the number of differentially expressed genes (DEGs) obtained with fold changes > 1.5 and 2.0 (*p* < 0.05) depending on the duration of SM treatment. We performed further integrated studies of the transcriptome data by analysis of identified DEGs.

Then, the microarray expression results were validated by a RT-PCR experiment for eight genes (up-regulated: *OSGIN1, IGFBP1, HMOX1, HERPUD1, GDF15, DDIT3*; down-regulated: *FASN, BRI3BP*) whose expression level was significantly altered in response to SM. As shown in Figure 1D, the expression trends of genes identified by microarray and RT-PCR correlated very well, thus confirming the reliability of obtained microarray data.

### The early effects of SM on transcriptome of KB-3-1 cells

In order to deeply understand the mechanism of SM action it is highly important to know its early effects on the transcriptome of tumor cells. Thus, in the first stage we analyzed DEGs detected during 1–4 h of SM treatment, consisting of 362 genes with 1.5-fold changes in expression (Figure 1E). As shown in Figure 1E, the response of tumor cells to SM was detected during the first hour of triterpenoid action. The number of DEGs identified at 1 h and 2 h time points was approximately constant and consisted of about 70 genes. Further incubation of cells with SM leaded to a subsequent twofold increasing of DEGs numbering up to 132 and 240 genes for 3 h and 4 h, respectively (Figure 1E). Among the detected DEGs, 17 genes were identified as common for the entire early period of SM’s action and included down-regulated DEGs controlling cellular metabolism (*MSMO1, ADSSL1, AGXT2L1, PFKFB4*), gene expression (*AHCY, APP*), nuclear organization (*HIST1H2BD, LBR*), intracellular transport (*RAB17, GOLIM4*), Ca^2+^ signaling (*CXCR4*), protein glycosylation (*DPM1*) and transport of γ-aminobutyric acid (*SLC6A12*). Common up-regulated DEGs were associated with stress-induced protein folding (*HSPA6*) and regulation of actin cytoskeleton organization (*CAPZB*).

**Figure 2:**
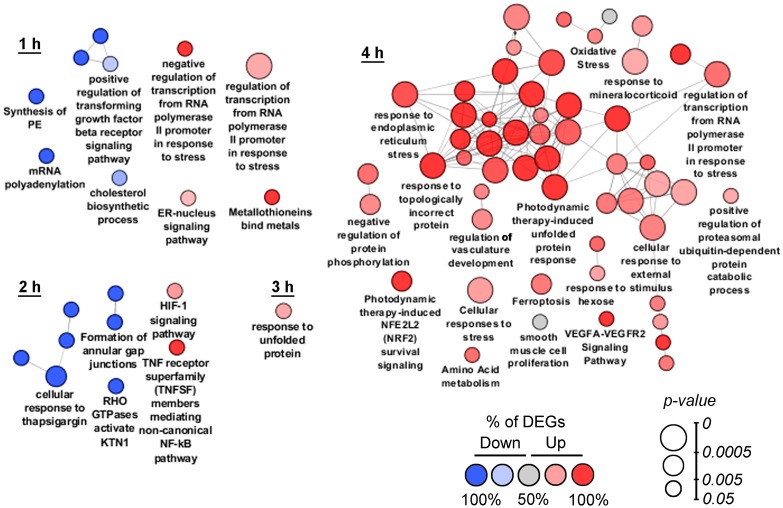
Functional analysis of DEGs detected at 1–4 h time points. Enrichment for Gene Ontology (biological processes), KEGG, REACTOME and Wikipathways terms were performed using the ClueGO plugin in Cytoscape. Functionally grouped networks with terms as nodes were linked based on their kappa score. The labels of the most significant terms per groups are shown. The size and color of nodes represent the term enrichment significance and the percentage of down- and up-regulated DEGs (fold change > 1.5) in the functional term, respectively. Only pathways with *p* < 0.05 after Bonferroni step down correction for multiple testing were included in the networks. Functionally related groups partially overlap.

In order to deeply understand the intracellular processes stimulated by SM treatment, functional analysis of DEGs was performed by using the ClueGO tool, being able to group and merge similar terms from Gene Ontology, KEGG, WikiPathways and REACTOME databases and therefore yield more authentic results in comparison with ordinary annotation sources [17]. Obtained ClueGO networks are shown in Figure 2. It was found that cellular stress, induced by SM in the early stages, was generally associated with its effect on the ER and the main intracellular events had developed by 4 h of SM treatment.

At the 1 h time point, SM suppressed genes involved in the biosynthesis of cholesterol (*INSIG1, LBR, MSMO1*) and lipids (*ETNPPL, LPIN1*), occurring in the ER, and modulated the ER-nucleus signaling pathway (*ATF3, DDIT3, INSIG1*) (Figure 2, 1 h). SM-induced stress was also accompanied by modulation of transcription (up-regulation of *ATF3, EGR1, DDIT3, CITED2* and *DNAJB1*; down-regulation of *ELOC* and *PSMA2*), suppression of mRNA polyadenylation (*APP, CPSF2, RNF20*) and TGF-β receptor signaling (*DAB2, CITED2, SDCBP*). Observed positive regulation of genes *MT1E*- and *MT1X-*encoded metallothioneins in the SM-treated samples can be explained by their probable direct interaction with SM. It is known that these cysteine-rich proteins can bind electrophiles by thiol groups [18]; cyano-enone triterpenoids in turn were shown to be able to readily react with thiols [19]. Unique DEGs, identified at the 1 h time point, showed high diversity and mainly included down-regulated genes associated with modulation of expression (*CDC14B, RNF20, SS18*), RNA processing (*CPSF2, HNRNPK, RRM2, UTP6*), cytoskeleton and membrane organization (*SYNE2, EHD1*), cell motility (*CRTAP, SUMF2, GNF, EPCAM*), mitochondrial transport (*SFXN1*), Ca^2+^ homeostasis (*PPP3R1*) and glycolysis (*ENO3*). Unique up-regulated DEGs at the 1 h time point included *CYP1B1, SLC25A10, PTPRU* and *MT1E*, related to drug metabolism, mitochondrial transport, cell adhesion and oxidative stress, respectively.

Gene set enrichment analysis of DEGs, identified after 2 h of SM treatment, confirmed the ER-targeted effect of SM. We found that “Cellular response to thapsigargin” (a known ER stress inducer) was one of the significantly changed terms at this time point. Other dysregulated pathways, associated with the ER, were linked with *KTN1*, encoded kinectin 1, an essential anchor for kinesin-driven vesicle motility, localized on ER cisternae [20]; and activation of cytoprotective HIF-1 and NF-kB signaling pathways, which are known to trigger in response to ER stress inducers [21, 22] and chemotherapeutic agents [23, 24], including triterpenoids [25, 26]. Moreover, NF-kB could play an important role in ER stress-induced death of CC cells – inhibition of NF-kB have been shown to effectively suppress autophagy and apoptosis of the cells, triggered by ER stress inducer brefeldin A [27]. The effect of SM at the 2 h time point was also accompanied by the suppression of genes *ACTG1* and *DAB2*, encoded γ-actin and clathrin adaptor protein, which is involved in clathrin-mediated endocytosis of gap junctions. At this time point, unique identified DEGs mainly include down-regulated genes associated with cytokinesis (*CENPF, KIF5D, SET11*), cytoskeleton organization (*MKLN1*), transport of cholesterol (*OSBPL9*), RNA decay (*UPF2*) and glutamate biosynthesis (*GLS*). Unique up-regulated DEGs at the 2 h time point are involved in transcription suppression (*COBRA1*), translation (*RPS6KB2*) and apoptosis induction (*LTBR*).

At the 3 h time point (Figure 2, 3 h) we identified high enrichment in genes associated with unfolded protein response (UPR), an intracellular signaling pathway performing homeostatic control of protein folding [28]. Induction of UPR-related genes by SM was in agreement with published data – previously, UPR-inducing activity was shown for different PTs [29–31]. Unique DEGs, identified at the 3 h time point, are mainly related to cellular signaling (receptors/G proteins (*IL17RD, GNB4, GNG12, GRIPAP1, QRFPR*), ionic channels (*CATSPER2, CHRNA5, FAM115C, MAGT1*), transcription regulators (*CREB1, GINS4, LRRFIP1, MBTD1, XPNPEP3, ZNF14, ZNF69, ZNF394, ZNF483, ZNF549, ZNF577, ZNF682*)), DNA repair (*C8orf45, DEM1, FAM175A, TRIM29, XRCC2*), lipid metabolism (*ACER3, HSD17B7, PTGR2, PTPLB, SPTLC1*), intracellular transport (*BLZF1, C15orf63, CHMP5, CLPTM1*), cytoskeleton organization (*C11orf63, C1orf222, MYO3B, ZYX*), nuclear structure and functions (*LMNA, PHAX, RCC1*), ubiquitin-dependent protein degradation (*FAM63A, PJA2, USP49*) and regulation of cell death (*APOPT1, BAX, CCND1, RASSF6, TAGLN2*).

Functional annotation of DEGs identified during 4 h of SM exposure revealed ER stress as a central intracellular event – the majority of obtained terms at this time point were mainly associated with this process (Figure 2, 4 h). Detected ER stress-related DEGs encoded molecular chaperones (*DNAJB9, FKBP14, HSPA5, HYOU1, SDF2L1*), proteins of ERAD machinery (*HERPUD, SYVN1, TRIM13*), regulators of transcription (*ATF3, CEBPB, DDIT3, JUN, XBP1, APP, IGFBP1, SESN2, TRIB3*), Ca^2+^ homeostasis (*STC2, SEC61A1*), thiol metabolism (*CHAC1, CTH, PDIA4*) and protein phosphorylation (*FAM129, PPP1R15A*). SM-induced cellular stress is accompanied by the up-regulation of genes controlling ubiquitin-dependent protein degradation, promoting removal of unfolded proteins from ER lumen, and cytoprotective Nrf2 signaling. These results correlated well with published data – on the one hand, the Nrf2 pathway was shown to be often activated by ER stress [32], on the other hand, cyano enone-bearing triterpenoids are known Nrf2 inducers (0.1 μM, U937 leukemia cells (0.5–8 h); hereinafter the treatment conditions by SM’s analogs leaded to mentioned activity will be indicated in the brackets) [16]. Identified high enrichment of “oxidative stress” could be also caused by ER stress as a result of Ca^2+^ leakage from the ER and its accumulation in the mitochondrial matrix or redundant ROS synthesis during protein folding [21]. Moreover, CDDO-Me, an SM analog, was shown to induce ROS generation in different tumor cell lines (1.25–2.5 μM, LNCaP/PC-3, OVCAR-5/MDAH 2774 and MiaPaCa-2/Panc-1 human prostate, ovarian and pancreatic cancer cells, respectively (1-2 h)) [33–35]. Up-regulated DEGs at 4 h time points are also involved in amino acid metabolism, which is known to be sensitive to many cellular stresses [36], ferroptosis, a ROS-dependent form of regulated cell death, which can be induced by some PTs [37, 38] and VEGF signaling, activated during ER stress [39]. High enrichment of DEGs related to the response to mineralocorticoids can be explained by the ability of SM to directly interact with mineralocorticoid receptors, like its parent compound glycyrrhetinic acid [40] or cause ligand-independent activation of the receptors, e. g. affecting RAC1 [41] – it was shown previously, that cyano enone-containing CDDO-Im can change RAC1 distribution on the cell membrane (1 μM; Rat2 fibroblasts (2 h)) [42].

### The late effects of SM on the transcriptome of KB-3-1 cells

**Figure 3:**
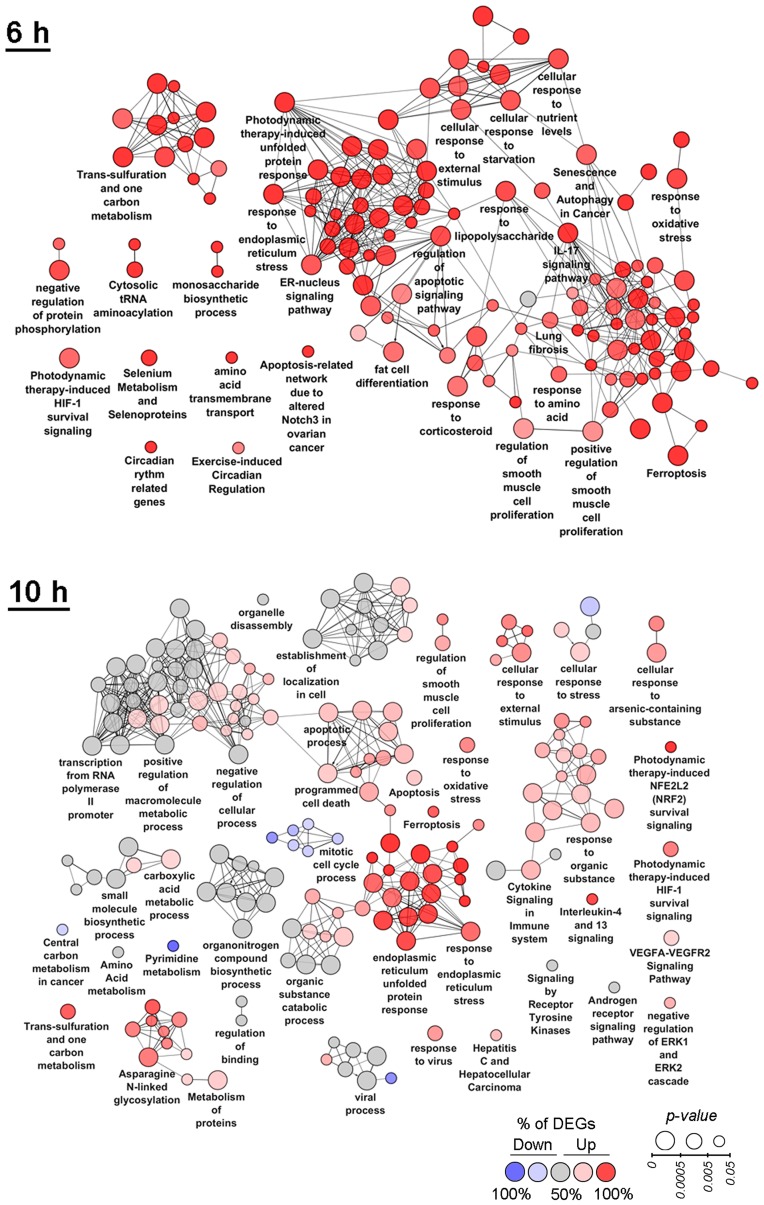
Functional analysis of DEGs detected at 6 h and 10 h time points. Enrichment for Gene Ontology (biological processes), KEGG, REACTOME and Wikipathways terms were performed using the ClueGO plugin in Cytoscape. Functionally grouped networks with terms as nodes were linked based on their kappa score. The labels of the most significant terms per groups are shown. The size and color of nodes represent the term enrichment significance and the percentage of down- and up-regulated DEGs (fold change > 1.5) in the functional term, respectively. Only pathways with *p* < 0.05 after Bonferroni step down correction for multiple testing were included in the networks. Functionally related groups partially overlap.

Functional annotation of DEGs, identified during the late phase (6–10 h) of SM treatment, still revealed the centrality of ER dysregulation in the complexity of SM-perturbed intracellular processes (Figure 3). Other highly enriched terms at these time points are associated with activation of the compensatory response of cells against SM action. Observed high up-regulation of apoptosis-related genes during this period can indicate insufficiency of cytoprotective mechanisms to overcome triterpenoid-induced stress.

Functional annotation of DEGs at the 6 h time point revealed high enrichment of autophagy that is in line with published data (Figure 3, 6 h) – it was shown previously that both ER stress and triterpenoids can cause autophagy [43–47]. At the 6 h time point up-regulated genes are involved in the response to lipopolysaccharide and IL-17 signaling that can indicate the activation of an inflammatory response, which is known to be highly interconnected with ER stress [21] and probably playing a pro-survival role – hyperexpression of IL-17 was shown to increase tumorigenicity of human cervical tumors in nude mice [48]. Other cytoprotective functional groups significantly changed by SM at the 6 h time point include the HIF-1 signaling pathway and the one carbon metabolism. The most highly enriched pathways also included lung fibrosis, selenium metabolism and selenoproteins and cytosolic tRNA aminoacylation, which can be associated with ER stress, according to published studies [49–51]. The effect of SM was also accompanied by the up-regulation of genes involved in the response to starvation, transmembrane transport of amino acids and monosaccharide biosynthetic processes, which could indicate the effort of cells to restore nutrient failures induced by stress. High enrichment of fat cell differentiation term in the SM-treated samples can be explained by the effect of SM on PPARγ, playing a key role in adipocyte differentiation [52] – previously, it was found that CDODA-Me had agonist activity on PPARγ (1-5 μM; SW480 colon cancer cells (20–22 h)) [53].

The negative effect of SM on KB-3-1 cell proliferation is significantly reinforced at 10 h of treatment (Figure 3, 10 h) – dysregulation of cell cycle process and a rise in the number of functional groups associated with programmed cell death are identified. ER stress was shown to remain a central event at this time point. Besides the terms directly indicating the activation of ER stress and UPR, a range of ER stress-associated pathways are significantly changed, such as the response to oxidative stress, asparagine N-linked glycosylation, cytoprotective Nrf2 and HIF-1 pathways and ER stress- and HIF-1-sensitive VEGFA-VEGFR2 signaling networks. The cellular stress response at the 10 h time point also includes activation of cytoprotective one carbon metabolism and disturbances in a range of other metabolic processes, including metabolism of pyrimidines, amino and carboxylic acids, small and macromolecules. The observed up-regulation of genes involved in cytokine signaling in immune system and IL-4/IL-13 signaling can be explained by the ER stress-induced inflammatory response and triggering of compensatory survival mechanisms, according to [21] and [54], respectively. Detected DEGs are also involved in signaling by receptor tyrosine kinases (RTKs) and the androgen receptor that is consistent with published data – previously, it was shown that PTs can inhibit RTK signaling pathways by direct interaction with the receptors and by suppression of their expression and phosphorylation status [4] and could directly bind to the androgen receptor, being its agonist or antagonist [55–57]. Negative regulation of ERK1/2, playing a critical role in cell proliferation and survival [58] and sensitivity to triterpenoid action [4], was also identified as an overrepresented category at the 10 h time point.

Among the identified highly enriched functional groups, three detected terms were also associated with antiviral response, including “viral process”, “response to virus” and “hepatitis C and hepatocellular carcinoma”, which contained DEGs involved in the regulation of innate antiviral immunity (*IFIT1, IFIT2, IFIT3, IFI16, ISG15, ISG20, LCN2, LGALS8*, etc.). Obtained data are in agreement with previously reported results – previously, we showed marked anti-influenza activity of SM (1 μM; influenza A-infected MDCK cells (24 h)) [12] and a range of PTs have been found to display anti-hepatitis C virus effects [59, 60].

### Analysis of co-expressed DEGs: clustering, functional annotation and search of probable master regulators

**Figure 4:**
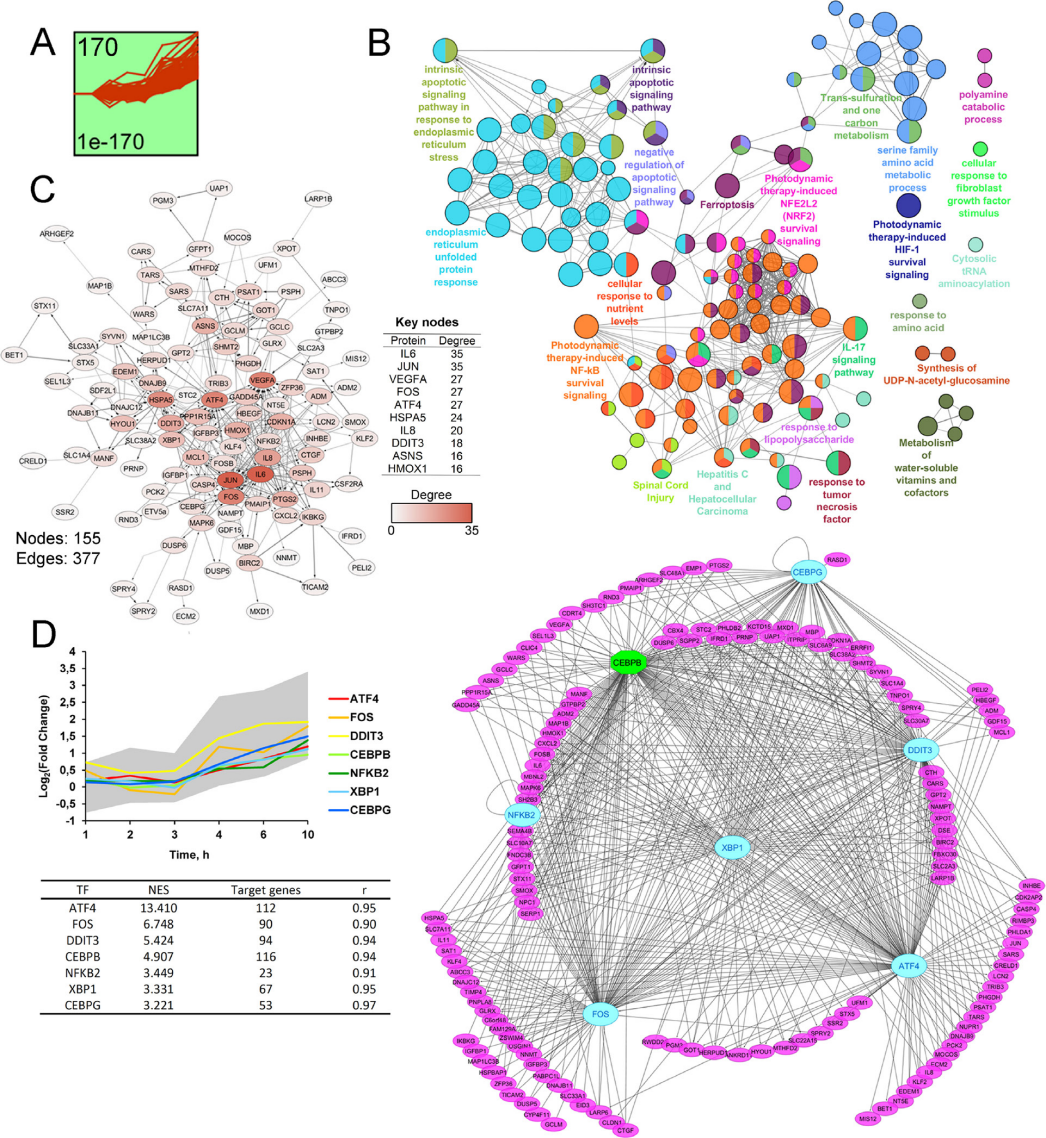
STEM clustering of DEGs revealed that co-expressed genes from Cluster 1 are mainly involved in regulation of ER homeostasis. (A) Expression profile of DEGs found in Cluster 1. The number in the top left corner represents the number of DEGs included in the cluster; the bottom left corner shows the *p*-value of the profile. X and Y-axes represent time points and relative gene expression levels, respectively. (**B**) The interaction network of significant terms enriched with DEGs from Cluster 1. Functional annotation was performed in ClueGO by using Gene Ontology (biological processes), KEGG, REACTOME and Wikipathways. The functionally grouped network is linked based on the kappa score of terms. The size and color of a node represents the term’s significance and its attachment to revealed groups. Only pathways with *p* < 0.05 after Bonferroni step down correction for multiple testing were included in the networks. (**C**) The protein-protein interaction network reconstructed with DEGs from cluster 1. The red gradient is proportional to the number of interactions (degree) of nodes with neighbors. The list of Top 10 DEGs with the highest degrees is shown in a table. Lines between nodes denote interactions between them (edges). (**D**) Potential transcription factors (TFs), regulating genes included in Cluster 1, were identified by iRegulon. TFs, characterized by normalized enrichment score (NES) > 3 and high correlation between TFs and target DEGs expression profiles (|r| > 0.9), are shown. The gray area and colored lines in the diagram represent the expression profile of DEGs and identified TFs, respectively. The right side of panel D shows the regulatory network of DEGs and TFs, marked by purple and blue/green colors, respectively. The blue oval and green octahedrons show TFs having upstream regulators/self-regulations or not in the network, respectively.

**Figure 5:**
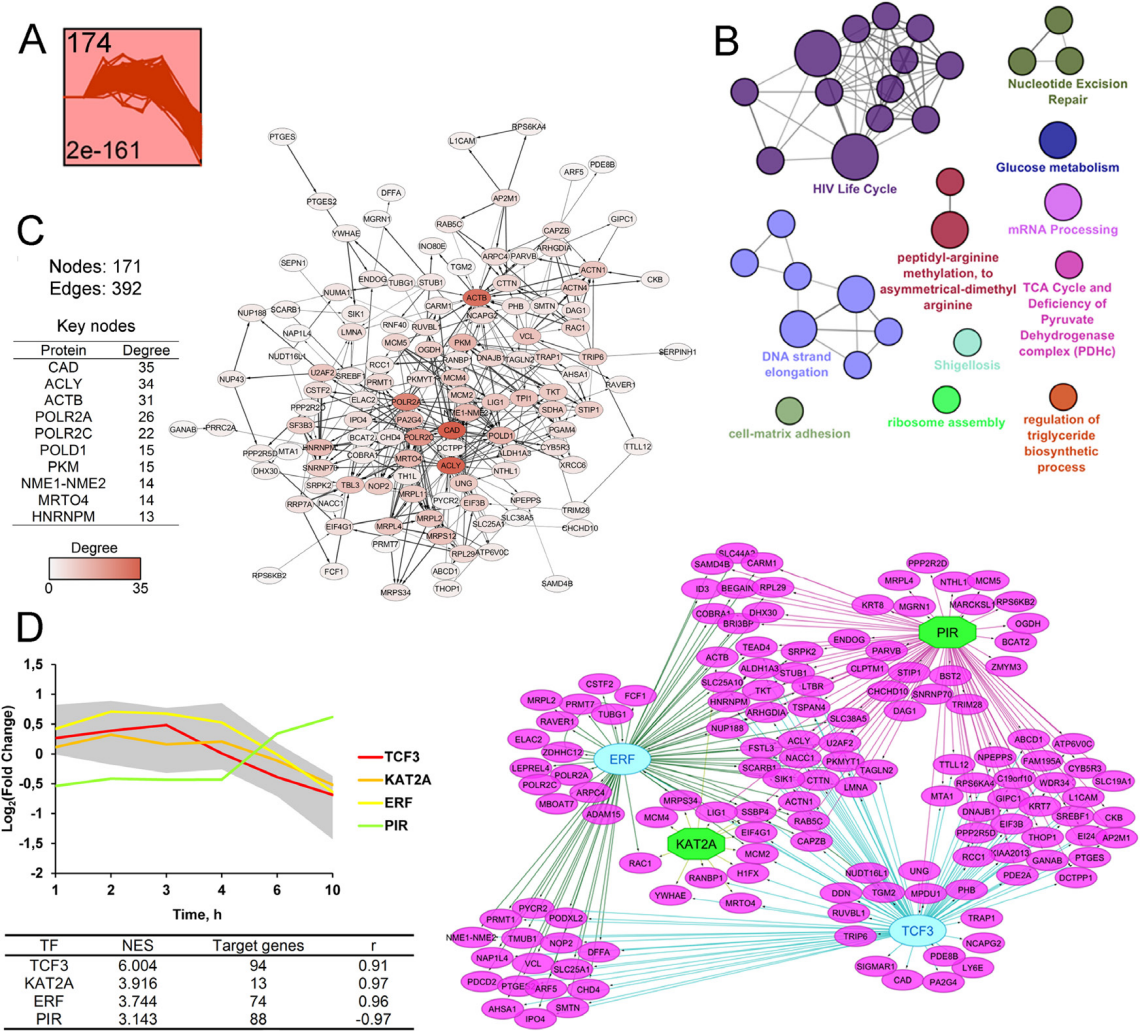
STEM clustering of DEGs revealed that co-expressed genes from Cluster 2 are mainly involved in regulation of cellular proliferation. (**A**) Expression profile of DEGs found in Cluster 2. The number in the top left corner represents the number of DEGs included in the cluster; the bottom left corner shows the *p*-value of the profile. The X and Y-axes represent time points and relative gene expression levels, respectively. (**B**) The interaction network of significant terms enriched with DEGs from Cluster 2. Functional annotation was performed on ClueGO by using Gene Ontology (biological processes), KEGG, REACTOME and Wikipathways. The functionally grouped network was linked based on the kappa score of terms. The size and color of nodes represent the terms’ significance and their attachment to revealed groups. Only pathways with *p* < 0.05 after Bonferroni step down correction for multiple testing were included in the networks. (**C**) The protein-protein interaction network reconstructed with DEGs from Cluster 2. The red gradient is proportional to the number of interactions (degree) of nodes with neighbors. The list of Top 10 DEGs with the highest degrees is shown in a table. Lines between nodes denote interactions between them (edges). (**D**) Potential transcription factors (TFs) regulating genes included in Cluster 2 were identified by iRegulon. TFs, characterized by normalized enrichment score (NES) > 3 and high correlation between TFs and target DEGs’ expression profiles (|r| > 0.9), are shown. The gray area and colored lines in the diagram represent the expression profile of DEGs and identified TFs, respectively. The right side of panel D shows the regulatory network of DEGs and TFs, marked by purple and blue/green colors, respectively. The blue oval and green octahedrons show TFs having upstream regulators/self-regulations or not in the network, respectively.

To better understand the key processes triggered by SM in KB-3-1 cells and reveal their master regulators sensible to SM action, time course clustering of gene expression profiles was performed by using the STEM clustering algorithm [61] (Figures 4, 5). This approach allows us to identify the populations of genes with similar expression patterns that are probably involved in the same cellular processes and could be hypothetically controlled by the same master regulators. Six significant clusters containing a total of 506 DEGs were found. The functional annotation of identified gene sets and a search of their probable upstream regulators were carried out. The results of the analysis for the most statistically overrepresented clusters 1 and 2 (*p* = 1E-170 and 2E-161, respectively, versus *p* = 5E-19 – 2E-5 for clusters 3–6) were shown in Figures 4 and 5. The acquired data for clusters 3–6 are presented in Supplementary Materials (Supplementary Figures 1–4).

### Co-expressed DEGs from Cluster 1 are mainly involved in regulation of ER homeostasis

Cluster 1 includes 170 up-regulated DEGs with a peak of expression at 10 h of SM treatment (Figure 4A). Functional analysis of genes from this cluster showed significant enrichment of the majority of terms and pathways mentioned above at 6 h and 10 h time points (Figure 4B). It was found that categories associated with ER stress were the most overrepresented – “ER unfolded protein response” and “intrinsic apoptotic signaling in response to ER stress” showed *p*-values equalling 4E-15 and 6E-10, respectively. DEGs from cluster 1 are also involved in the regulation of cell death, inflammation (response to LPS, IL-17 signaling pathway), cytosolic tRNA aminoacylation, polyamine catabolic process and Nrf2, NF-kB and HIF-1 survival signaling pathways, which can be also considered as ER stress-associated processes, according to published reports [21, 22, 32, 51, 62, 63]. Other highly enriched functional terms included “one carbon metabolism” and “response to fibroblast growth factor”, which are known to play a cytoprotective role [64]. Analyzed DEGs were also associated with response to TNF and spinal cord injury - the pathways, lying on the boundary between inflammatory response and cell death. The majority of DEGs, involved in the pathway “Hepatitis C and Hepatocellular carcinoma”, encode pro-inflammatory proteins (IL-6, IL-8, PTGS2), and, therefore, this term should be also considered as inflammation related. Other overrepresented categories are synthesis of UDP-N-acetyl-glucosamine, playing an important role in post-translational modification of proteins, and a range of metabolic pathways.

A further, protein-protein interaction (PPI) network with proteins encoded by DEGs from cluster 1 constructed by retrieving relatively high confident protein interactions (confidence score: 0.7) from the STRING database. PPI network, consisting of 111 nodes and 377 interactions (or edges), was obtained (Figure 4C). Subsequent ranking of nodes in terms of their level of interconnection in the PPI network was performed and key molecules, characterized by maximum number of edges, were identified (Figure 4C, table). In agreement with the previous report [65], such key nodes can be considered as probable master regulators of observed cellular processes. The top-10 of detected key nodes for cluster 1 includes subunits of stress-activated transcription factor AP-1 (JUN, FOS), pro-inflammatory and pro-angiogenic cytokines (IL-6, IL-8, VEGF), ER stress pathway regulators (HSPA5, ATF4, DDIT3, ASNS) and antioxidant protein HO-1. Obtained results agree well with published data and functional annotation of DEGs, mentioned above. Previously, significant up-regulation of AP-1 subunits and HO-1 was identified for cyano-enone-bearing triterpenoid CDDO-Im in HUVEC cells (0.2 μM; HUVEC (6 h)) [66]. Entry of cytoprotective AP-1, HO-1, pro-inflammatory and pro-angiogenic cytokines into the top list of key nodes probably indicated the triggering of a compensatory mechanism in response to SM-induced stress in KB-3-1 cells, which was in line with overrepresentation of survival signaling pathways, as defined above (Figures 2 and 3). Disclosure of central ER stress-associated factors HSPA5, ATF4, DDIT3 and ASNS as probable master regulators independently confirmed the ER-targeted effect of SM.

Based on the fact that co-expressed genes could be regulated by the common transcription factors (TFs), analysis of *cis*-regulatory elements within promoters of DEGs was performed by using the iRegulon tool [67] and candidate TFs, being hypothetically able to control expression of uploaded DEGs, were predicted (Figure 4D). Obtained TFs were further ranked by Normalized Enrichment Score (NES) and significantly enriched regulators with NES > 3 were selected. Then, according to [68], the Pearson correlation coefficient was calculated for all pair-wise comparisons of gene expression profiles between selected TFs and DEGs in order to identify the potential regulatory relationship. The relationship with r > 0.9 was considered as significant. In total, 6 TFs satisfying both NES and Pearson’s r thresholds, were identified and visualized with their target DEGs by Cytoscape (Figure 4D). Interestingly, the majority of revealed TFs (ATF4, DDIT3, CEBPB, XBP1, CEBPG) are involved in the regulation of UPR and ER stress [28, 69]. Promoter analysis, performed with the help of geneXplain platform [70, 71], also showed that 23 DEGs from cluster 1 can be regulated by NFKB2 that clearly conforms to high enrichment of NF-kB survival signaling, detected by us (Figure 2, Figure 4B). Observed overrepresentation of ER stress-associated TFs and, in addition, the involvement of ATF4 and DDIT3 into the Top-10 key nodes of the PPI network (Figure 4C) clearly shows centrality of dysregulation of ER homeostasis in SM-triggered intracellular perturbations.

### Co-expressed DEGs from Cluster 2 are associated with regulation of cellular proliferation

The second highly enriched cluster includes 174 DEGs characterized by progressive decline in expression level at 6–10 h time points (Figure 5A). Functional annotation of DEGs revealed dysregulation of major intranuclear pathways (Figure 5B), including nucleotide excision repair (*ACTB, INO80E, LIG1, POLD1, POLR2A, POLR2C, RUVBL1*), DNA strand elongation (*LIG1, MCM2, MCM4, MCM5, POLD1*), mRNA processing (*CSTF2, HNRNPM, POLR2A, PRMT1, SF3B3, SNRNP70, SRPK2, U2AF2*), cellular energetics, particularly glucose metabolism (*NUP188, NUP43, PKM, PPP2R5D, SLC25A1, SLC25A10, TPI1*) and TCA cycle (*ACLY, OGDH, SDHA*). Another overrepresented category is ribosome assembly (*DHX30, MRPL11, MRTO4, NOP2, RRP7A*) that is in line with the probable ER-targeted action of SM – previously, the inhibition of ribosome assembly and subsequent cancellation of translation were detected during ER stress [72]. DEGs from cluster 2 are also involved in the regulation of the actin cytoskeleton (*ACTB, ARPC4, CTTN, RAC1, VCL*) and protein methylation (*CARM1, PRMT1, PRMT7*), playing important roles in shigellosis and transcription regulation [73], respectively; triglyceride biosynthesis (*SCARB1, SIK1, SREBF1*) and cell-matrix adhesion (*ACTN1, ADAM15, BCAT2, CTTN, DAG1, L1CAM, RAC1, TRIP6, VCL*). Cluster 2 also includes DEG-encoded proteins exposed on the nuclear membrane (*NUP43, NUP188, RANBP1*) and intranuclear proteins (*LIG1, NELFB, NELFCD, POLR2A, POLR2C, RCC1, XRCC6*), being important regulators of the HIV-1 life cycle. Obtained results are consistent with published data – a range of PTs were previously shown to inhibit HIV-1 replication [74].

Next, the PPI network consisting of 124 nodes and 390 edges were reconstructed from analyzed DEGs (Figure 5C). Obtained nodes were further ranked in terms of their interconnection in the network and the Top-10 key nodes were identified (Figure 5C, table). Analysis revealed CAD, playing a crucial role in pyrimidine biosynthesis, as a node with the highest degree (degree = 35). The observed high degree of centrality of CAD was probably explained by the strong negative effect of SM on cell viability. Previously, high correlation between up-regulation of CAD and the cell proliferation level was detected [75]. A list of the obtained top key nodes also includes ACLY, a central enzyme in lipid biosynthesis, expression of which can be inhibited in response to triterpenoids [76] or cyano-enone-containing compounds [77]. Another nodal protein is beta actin (ACTB), displaying a high interconnection into the PPI network (degree = 31) (Figure 5C), which can be also considered as a probable primary protein target of cyano enone-bearing triterpenoids: previously, To and coworkers found that CDDO-Me can directly bind to ACTB and actin-related proteins (10 μM; Rat2 fibroblasts (2 h)) [78]. Other nodes with a high degree of centrality are subunits of polymerases II (POLR2A, POLR2c) and δ (POLD1) that play a key role in mRNA transcription and DNA repair, respectively; pyruvate kinase M2 (PKM) that supports cell proliferation by promoting NAPDH and other macromolecule production, NME1-NME2, which can modulate the activity of small GTPase, playing a pivotal role in the regulation of numerous cellular functions, MRTO4, a component of the ribosome assembly machinery, and HNRNPM, playing an important role in mRNA processing.

Promoter analysis revealed four TFs, which probably regulate the expression of DEGs from cluster 2 (Figure 5D). Revealed TFs include TCF3, KAT2A, ERF and PIR involved in the regulation of cellular proliferation. It was found that TCF3, displaying the highest NES value, was up-regulated in cervical cancer and its down-regulation significantly inhibited the growth and invasion of cervical carcinoma cells [79]. KAT2A and PIR was shown to display NF-kB repressor and co-activator functions, respectively [80, 81], and therefore can be related to compensatory survival mechanism triggering in response to SM treatment.

As can be seen from the analysis, DEGs from cluster 2 (Figure 5B) are associated with significantly less functional groups as compared with genes from cluster 1 (Figure 4B). However, detected pathways were characterized by high heterogeneity and the identification of central processes among them is not so obvious as in the case of cluster 1. Pathway analysis of key nodes and TFs also revealed high diversity of their functions. Observed dysregulation is probably linked with the inhibition of different vital cellular processes due to the triggering of cell death by SM treatment.

Besides highly overrepresented clusters 1 (*p* = 1E-170) and 2 (*p* = 2E-161), STEM clustering also revealed four minor clusters 3–6 displaying significantly higher *p*-values (*p* = 5E-19 – 2E-5) (Supplementary Figures 2–4). DEGs from clusters 3–5 are characterized by similar patterns of expression with an increase of a fold change at 6–10 h time points, whereas cluster 6 includes DEGs that reach a peak of expression at 3 h. According to the STRING database, proteins encoded by genes from clusters 3–6 display a low level of interconnection (Supplementary Figures 2–4). Functional enrichment analysis of DEGs from minor clusters showed their association with the inhibition of cell proliferation, innate immune response, protein export (cluster 3), ER stress, Golgi-related intracellular transport and degradation of organelles (cluster 4), interferon signaling, inhibition of virus replication, angiogenesis (cluster 5) and muscle tissue development (cluster 6).

### Reconstruction and analysis of the PPI network

**Figure 6:**
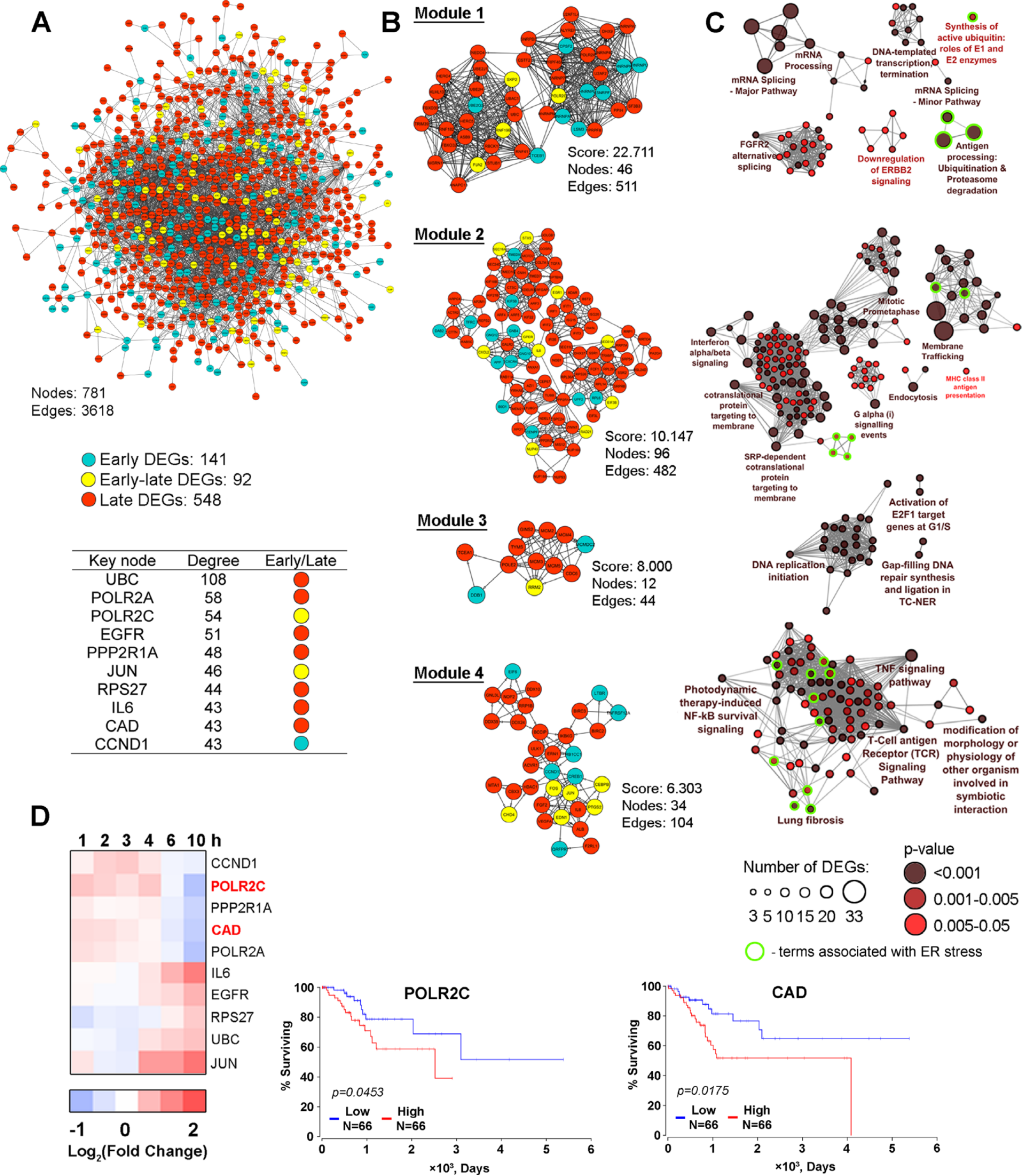
The protein-protein interaction network. (**A**) The protein-protein interaction network reconstructed with DEGs (fold change > 1.5) identified in all investigated time intervals (1–10 h). Blue and red nodes show DEGs detected during the early (1–4 h) or late (6–10 h) phase of SM treatment, respectively. Yellow nodes show genes that changed their expression level regardless of the duration of SM treatment (early-late DEGs). Node degree characterizes the number of interactions of nodes with neighbors in the PPI network. The list of Top 10 key nodes is shown in a table. (**B**) Module analysis of the PPI network by using the MCODE clustering algorithm. Only modules with a score > 6 are shown. The color of nodes indicates the belonging of genes to early, early-late or late responding DEGs groups. (**C**) Functional annotation of DEGs from modules performed by the ClueGO plugin. The functionally grouped network is linked based on the kappa score of terms. The size and color intensity of nodes represent the number of DEGs in the terms and the term’s significance, respectively. Only pathways with *p* < 0.05 after Bonferroni step down correction for multiple testing were included in the networks. Functional groups associated with ER stress were marked by green circles. (**D**) POLR2C and CAD expression are negatively associated with the overall survival time of CESC patients. The heat map shows the changing of the expression of key nodes in SM-treated KB-3-1 cells in comparison with untreated cells. Overall survival curves for CESC patients were constructed based on TCGA data by using OncoLnc.

Next, in order to evaluate the level of interconnection of all modulated DEGs (fold change > 1.5) and independently reveal the major pathways dysregulated by SM, we reconstructed the PPI network from proteins encoded by DEGs, using STRING. The PPI network consisting of 781 nodes and 3618 edges was created (Figure 6A). As shown in Figure 6A, the main components (70%) of the obtained PPI network are proteins encoded by genes that changed their expression level during the late phase of SM treatment (6–10 h). Proteins encoded by early responding DEGs (1–4 h) or DEGs with changed expression during both the early and late phases of SM treatment (early-late DEGs) made up 18% and 12% of total DEGs in obtained the PPI network, respectively.

In order to make our study more informative, the search of highly connected functional modules in the PPI network was performed using the MCODE plugin. We identified four modules that are graphically represented in Figure 6B. Obtained modules are characterized by similar content of early, early-late and late DEGs as the whole PPI network. Further functional enrichment analysis of genes composing of each module revealed a high diversity of overrepresented categories, including ER stress-associated pathways identified in modules 1, 2 and 4 (Figure 6C, green outlines).

It was shown that 46 DEGs included in the most interlinking module 1 are involved in DNA transcription and mRNA splicing, already mentioned above, and the down-regulation of HER2 signaling, which is known to participate in pathologic growth of cervical carcinomas [82]. Four terms associated with ubiquitin-dependent protein degradation were identified as ER stress-related (Figure 6C, green outlines). Module 2 includes 96 genes that are mainly involved in intracellular transport (membrane trafficking, endocytosis, MHC class II antigen presentation), mitosis and modulation of PKA and interferon α/β signaling pathways. A range of minor terms in module 2 are found to be related to ER stress, notably ER unfolded protein response, activation of chaperones by ER stress master-regulators IRE1α and XBP1 and ER to Golgi transport (Figure 6C, green outlines). Twelve DEGs from module 3 are involved in regulation of S phase of cell cycle and DNA reparation. Module 4 includes 34 genes, being associated with TNF and TCR signaling pathways and also involved in ER stress-related major (NF-kB survival signaling, lung fibrosis) and minor (AP-1 and HIF1α survival signaling, apoptosis, autophagy and inflammation) terms (Figure 6C, green outlines). Thus, PPI network analysis independently confirmed the data obtained previously by pathway analysis of DEGs and STEM clustering and clearly shows the capability of SM to dysregulate ER homeostasis.

Next, we ranged the nodes included in the PPI network according to their levels of interconnection and revealed key molecules that can play central regulatory functions in the processes induced in cells by SM (Figure 6A). It was found that a half of revealed key nodes were already identified by us as probable master regulators – subunit of AP-1 transcription factor JUN and pro-inflammatory cytokine IL-6 were characterized by a high degree of centrality in the PPI network related to STEM cluster 1 (Figure 4C); POLR2A, POLR2C and CAD, playing important roles in mRNA transcription, DNA repair and cell proliferation, respectively, were detected as key nodes in STEM cluster 2 analysis (Figure 5C). UBC, encoded ubiquitin C, was found to display the highest degree score (Figure 6A, table) that can be explained by its ability to regulate a wide variety of cellular processes, including protein degradation, cell cycle progression, DNA repair, endocytosis and a range of signaling pathways [83], detected as SM-sensitive (Figures 2–6). Another nodal protein is EGFR, an epidermal growth factor receptor that plays an important role in the proliferation of tumor cells. The high degree of centrality of EGFR correlated with the ability of cyano enone-bearing triterpenoids to interact with this protein. Previously, we found that SM is unambiguously docked to EGFR and probably inhibits its activity [12]; Liby and colleagues showed that CDDO-Im can directly bind to the receptor (3 μM; PDA 4964 pancreatic cancer cells (1 h)) [84]. The Top-10 hub nodes also include PPP2R1A, an integral regulator subunit of protein phosphatase PP2A (Figure 6A), a participant of many signal transduction pathways. The high degree score of PPP2R1A indicates the probable involvement of PP2A in tumor cell responses to cyano enone-containing triterpenoids, which is consistent with published data. Previously it was found that the expression and phosphatase activity of PP2A were inhibited by CDDO-Me in LNCaP human prostate cancer cells (1.25-5 μM (20 h)) [85]. Identification of ribosomal protein RPS27 as a nodal molecule can be explained by its extraribosomal functions associated with regulation of cell proliferation [86, 87]. The list of revealed Top-10 key nodes was completed by cyclin D1 (CCND1), a key regulator of G1 to S phase progression, being sensitive to triterpenoid action [4].

As the next step of the study, we analyze whether the revealed key nodes are involved in cervical carcinoma progression. In consideration of the tight interconnection between tumor progression and disease outcome, we tried to find associations between the expression of key nodes modulated by SM and the survival rate of patients with cervical squamous cell carcinoma and endocervical adenocarcinoma (CESC) using TCGA clinical data. We revealed that two of ten hub nodes, POLR2C and CAD, are associated with bad prognoses for CESC patients: we found a shorter overall survival time in patients with higher expression levels of these genes (Figure 6D). Analysis showed that SM inhibits expression of POLR2C and CAD at the late phase of KB-3-1 cell treatment (Figure 6D, heatmap). Thus, the data confirm the probable involvement of these hub genes in the regulation of cell response to SM.

### Effect of SM on the expression of genes associated with CESC growth

**Figure 7:**
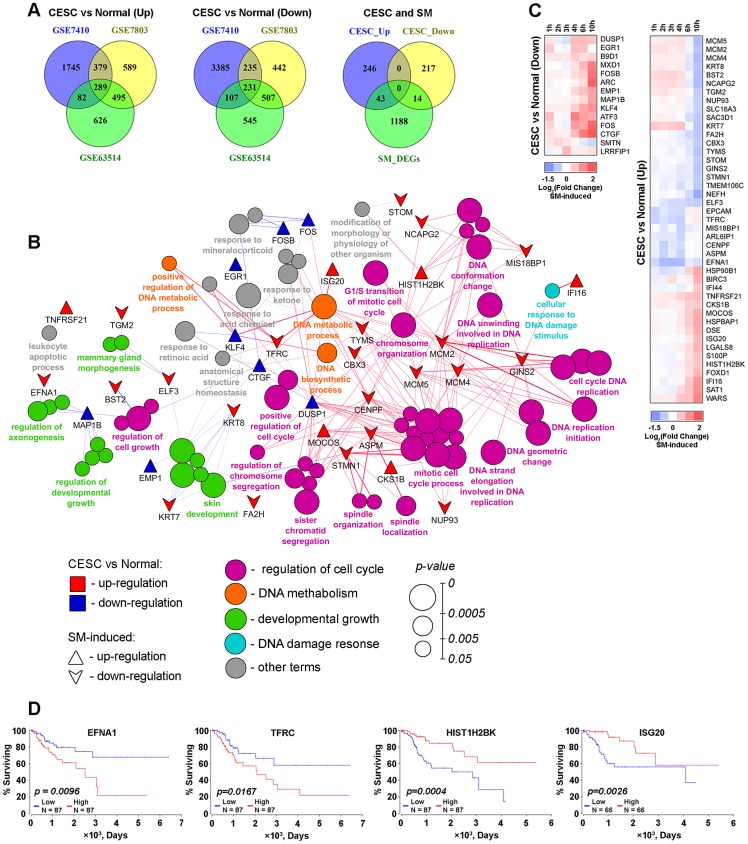
SM reversed expression of genes playing a crucial role in the vitality of cervical cancer cells. (**A**) Venn diagrams of genes differentially expressed in CESC in comparison with normal cervix (DEGs^CESC^) identified by reanalysis of GSE7410, GSE7803 and GSE63514 datasets. From left to right: up-regulated DEGs^CESC^ (fold change > 1.5), down-regulated DEGs^CESC^ (fold change < 1.5) and venn diagram illustrating intersection of identified overlapped up- and down-regulated DEGs^CESC^ (CESC_Up and CESC_Down, resprectively) with DEGs between SM-treated and untreated KB-3-1 cervical carcinoma cells (SM_DEGs, |fold change| > 1.5). (**B**) Functional annotation of overlapped 289 up- and 231 down-regulated DEGs^CESC^ performed by the CluePedia plugin. Rings show overrepresented functional terms. Terms are functionally grouped based on shared proteins (kappa score > 0.7) and their colors represent different functional classes. The size of nodes indicates the degree of significance. Within each group, the most significant term defines the name of group. Triangles and downward arrows represent up- and down-regulated DEGs between SM-treated and untreated cells, respectively, overlapping with DEGs^CESC^. Green and blue colored of triangles and arrows show up- and down-regulation of the genes between CESC vs Normal cervix, respectively. (**C**) Heat map showing the changing of expression of DEGs between SM-treated and untreated cells. CESC vs Normal (Up) and CESC vs Normal (Down) represent that marked genes are up- or down-regulated (fold change > 1.5) in CESC in comparison with healthy cervix. (**D**) High expression of EFNA1 and TFRC and low expression of HIST1H2BK and ISG20 are negatively associated with overall survival time for patients with CESC. Overall survival curves were constructed based on TCGA data by using OncoLnc.

In order to assess the effect of SM on the expression of key genes responsible for the high proliferation rate of CC cells, changes in gene expression between CESC tissues and their normal counterparts were firstly identified by re-analysis of microarray datasets from GSE7410, GSE7803 and GSE63514 using the GEO2R tool (Figure 7). Venn diagram analysis of revealed DEGs (fold change > 1.5, *p* < 0.001) were further performed (Figure 7A). A total of 289 up-regulated and 231 down-regulated DEGs, being common for all analyzed GEO datasets, were identified. Functional analysis of the obtained 520 overlapped DEGs revealed high enrichment of terms mainly associated with regulation of the cell cycle, developmental growth, DNA metabolism and DNA damage response (Figure 7B). We suppose that identified genes determine the high growth rate of CESC in comparison with healthy cervix tissue and could be considered as probable therapeutic targets in cervical carcinoma.

Next, we questioned whether SM could modulate the expression of identified genes. Matching of the lists of CESC-associated DEGs with genes responding to SM treatment revealed 43 up- and 14 down-regulated overlapped DEGs (Figure 7, right venn diagram). Interestingly, SM mainly inverts expression of these genes – 13 of 14 genes, down-regulated in CESC, were up-regulated after SM treatment; expression of 27 of 43 genes, up-regulated in CESC, were suppressed by SM (Figure 7C). Moreover, we found that a range of these genes were associated with the overall survival of CESC patients (Figure 7D). Kaplan-Meier plots showed that high expression of EFNA1 and TFRC and low expression of HIST1H2BK and ISG20 are correlated with poor prognosis (log-rank test *p*-value 0.0096–0.0167). The ability of SM to inversely down-regulate EFNA1 and TFRC and up-regulate HIST1H2BK and ISG20 could illustrate a probable importance of these genes in SM-induced inhibition of growth of cervical carcinoma cells.

### SERCA and GRP94 are probable primary targets of SM

**Figure 8:**
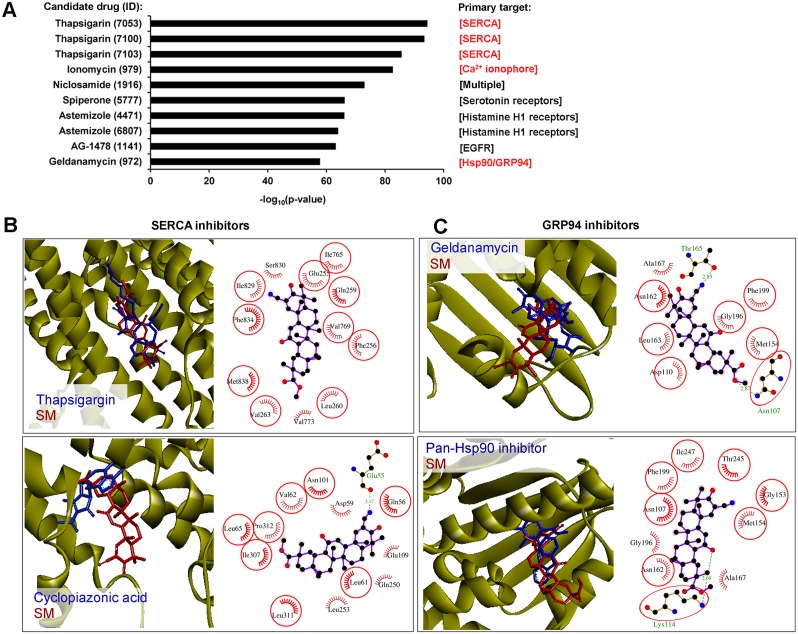
SERCA and GRP94 could be considered as probable direct targets of SM. (**A**) CMap analysis found a strong similarity between the SM-induced gene expression signature and those induced by known ER stress activators. The diagram shows the Top 10 candidate drugs from CMap analysis that displayed similar effects on the transcriptome of tumor cells to that of SM. Primary targets of the drugs, dysregulation of which caused ER stress, are marked in red. CMap analysis performed on ToppGene Suite (https://toppgene.cchmc.org), up- and down-regulated DEGs (fold change > 1.5), identified in KB-3-1 treated by SM for 10 h, were used as the input query. (**B**) The mode of binding of SM to SERCA. Stereo presentation of docked poses of SM in SERCA, superimposed on thapsigargin and cyclopiazonic acid-bound structures (PDB IDs: 2AGV and 1OA0, respectively), was drawn by BIOVIA Discovery Studio. Structures of SERCA inhibitors and SM are depicted by blue and red sticks, respectively. 2D representation of docked poses of SM in SERCA is created by LigPlot+. The combs and green dashed lines represent hydrophobic interactions and hydrogen bonds, respectively. Common residues, interacting with both inhibitors and SM, are highlighted in red circles. Color coding of atoms in ball format: black – carbon, blue – nitrogen and red – oxygen. (**C**) The mode of binding of SM to GRP94. Stereo presentation of docked poses of SM in GRP94, superimposed on geldanamycin and methyl 2-[2-(2-benzylpyridin-3-yl) ethyl]-3-chloro-4,6-dihydroxybenzoate (Pan-Hsp90 inhibitor)-bound structures (PDB IDs: 2EXL and 6ASQ, respectively), were drawn by BIOVIA Discovery Studio. Structures of GRP94 inhibitors and SM are depicted by blue and red sticks, respectively. The 2D representation of docked poses of SM in GRP94 was created by LigPlot+.

To further evaluate whether the responses of tumor cells to SM are similar to those of known therapeutic drugs, Connectivity Map (CMap) analysis was performed. The DEGs with fold change >1.5 were selected and used as the input query into CMap annotation conducted via ToppGene Suite (https://toppgene.cchmc.org). The ranking of candidate drugs was established based on *p*-value and the top ten highest ranked compounds are shown in Figure 8A. Interestingly, CMap analysis revealed a similarity of the effect of SM on transcriptome of tumor cells with the effects of known ER-stress inducers – thapsigargin, ionomycin and geldanamycin [88–90] that independently corroborate our hypothesis about the ER-targeted action of SM. Other candidate agents also included multiple pathway inhibitor niclosamide, modulators of serotonin and histamine H1 receptors spiperone and astemizole, respectively, and EGFR inhibitor AG-1478. In accordance with the high enrichment of ER stress-associated terms after SM treatment mentioned previously, we further concentrated our attention on the drugs affecting ER homeostasis.

Thapsigargin and ionomycin are known modulators of intracellular Ca^2+^ balance, inhibiting sarcoplasmic/endoplasmic reticulum Ca^2+^ ATPase (SERCA) activity and showing Ca^2+^ ionophoric effect, respectively. As a result of their action, an increase of cytoplasmic [Ca^2+^] and subsequent activation of UPR were observed [89, 91]. It was shown that triterpenoids could also act on Ca^2+^ homeostasis – SM analogs CDDO and CDDO-Me were found to induce the release of intracellular calcium stores into the cytoplasm of tumor cells (1–10 μM (COLO 16 skin carcinoma cells (1 h)) and 1.5 μM (MDA-MB 435 breast cancer cells (2–6 h)), respectively) [92, 93]; another semisynthetic triterpenoid LAFIS13 and a range of triterpenoid saponins can directly bind to SERCA [94] and effectively inhibit its activity [95, 96]. Geldanamycin is a selective inhibitor of Hsp90 and ER Hsp90 paralog Grp94, directly binding to the N-domain and competing for the ATP binding site of the proteins [97]. By inhibition of these chaperons, geldanamycin dysregulates protein folding in the ER and, as a result, induces the ER stress pathway [90]. It was recently found that CDDO-Me can also directly interact with Hsp90 and suppress its functions (1.25–2.5 μM; HO8910/ SKOV3 ovarian cancer cells (3-24 h)) [98], however, its effect on Grp94 activity had not been evaluated. In virtue of drug similarities, identified by CMap analysis (Figure 8A), the known ability of triterpenoids to effect on Ca^2+^ homeostasis and Hsp90 activity and the high homology between Hsp90 and Grp94, we supposed that SERCA and Grp94 could be considered as potential molecular targets of SM.

In order to assess the ability of SM to bind to SERCA and Grp94, molecular docking simulations were performed. Our results showed that the hydrophobic scaffold of SM bridges the M3 and M7 transmembrane helices of SERCA, similar to thapsigargin (Figure 8B, upper panel). Though SM does not form hydrogen bond interactions with SERCA, triterpenoid interacts with hydrophobic residues Phe256, Ile765 and Phe834, being the most important for thapsigargin inhibitory activity [99]. Further, we docked SM with the binding site of another SERCA inhibitor – cyclopiazonic acid (CPA) (Figure 8B, lower panel) and showed that the SM binding pocket is similar to that of CPA. The triterpenoid core of SM is stabilized by the hydrophobic residues Gln56, Asp59, Leu61, Val62, Leu65, Asn101, Glu109, Glsn250, Leu253, Ile307, Leu311 and Pro312, the majority of which participate in the binding of CPA. The CN group at the C2 position of SM forms a strong hydrogen bond with the backbone of Glu55 (3.17 Å).

The interactions between SM and Grp94 were explored by using two Grp94 crystal structures 2EXL and 6ASQ in the binding sites of known Hsp90/Grp94 inhibitors geldanamycin and methyl 2-[2-(2-benzylpyridin-3-yl) ethyl]-3-chloro-4,6-dihydroxybenzoate, respectively. The docking model of SM in Grp94 showed that triterpenoid can snugly fit into the active site of the chaperone in positions very close to that of both inhibitors (Figure 8C). In the case of 2EXL, the CN group at the C2 position of SM forms a hydrogen bond interaction with Thr165 (2.85 Å), whereas the triterpenoid core occupies the hydrophobic pocket (Asp110, Met154, Asn162, Leu163, Gly196, Phe199) in the binding site (Figure 8C, upper panel). In the case of 6ASQ, the ketone group at the C12 position of SM interacts with the side chain amino group of Lys114 to form a hydrogen bond (2.68 Å) and triterpenoid is also stabilized by hydrophobic residues Asn107, Met154, Gly153, Asn162, Ala167, Gly196, Phe199, Thr245 and Ile247 (Figure 8C, lower panel). Obtained data indicate that SM could target SERCA and Grp94.

## DISCUSSION

PTs can be considered as a promising source of novel antitumor agents, being able to affect multiple targets in tumor cells [4]. Despite the large effort devoted to the evaluation of antitumor activity of PTs in different tumor cell lines, today there are no full understandings of the molecular mechanism of action of these compounds in cervical carcinoma cells. Analysis of published data in this field revealed the high interest of researchers in the induction of programmed cell death by triterpenoids, however, master regulators of such effects and, moreover, the primary targets of triterpenoids in CC cells remain unknown (Supplementary Table 1).

In detail, natural PTs were found to inhibit the proliferation of a range of CC cell lines, including both human papilloma virus-positive [100–109] and -negative [106, 109] cells. To reveal the probable mechanism of such inhibitory effects, authors mainly concentrated on the analysis of pro-apoptogenic activity of the compounds by using a range of standard assays. Indeed, it was shown that triterpenoids triggered apoptosis in CC cells through both intrinsic and extrinsic pathways, as evidenced by the release of cytosol cytochrome C [101], increase of Bax/Bcl-2 ratio [100, 101, 104, 106–108], dissipation of mitochondrial membrane potential [100, 101, 105] and activation of caspase-9 [104, 105, 107] in the case of intrinsic apoptotic perturbations, and an increase of the expression of Fas [107, 109] and activation of caspase-8 [107, 109, 110] in the case of the extrinsic pathway. Moreover, natural PTs were able to arrest the cell cycle of CC cells at G_0_/G_1_ [100, 105], S [108] or G_2_/M [102] phases depending on the considered compounds. However, more detailed analysis of their mechanism of action preceding the triggering of apoptosis in considered tumor cell lines has not been published yet. Only three author groups reported involvement of the modulation of the PI3K/Akt signaling axis or NF-kB and ERK1/2 in the pro-apoptogenic activity of betulinic [105] or ursolic [104, 106] acids, respectively.

Semisynthetic triterpenoids were also investigated in relation to the viability of CC cells (Supplementary Table 1). The works in this field could be conventionally divided into two main groups by the type of transformation: introduced into triterpenoid scaffold cyano enone pharmacophore [11, 111–116], or other substituents [117–120]. According to the transformation type, the depth of the studies was found to be significantly different. In the case of derivatives that did not contain the cyano enone pharmacophore, CC cell lines were used only for the evaluation of cytotoxicity of compounds during screening assays [117–120]. In the case of cyano enone-bearing triterpenoids, researchers used CC cells not only for the estimation of the antiproliferative activity of compounds (IC_50_ = 3–50 μM; see Supplementary Table 1) [121, 122], but also for uncovering their effect on intracellular signaling pathways [111–116]. However, CC cells were used here mainly as additional models to perform the luciferase reporter [111, 112] and transactivation [113] assays and to estimate the effect of the compounds on a range of enzymes [114, 115], whereas the main part of the studies was carried out on other tumor cell types. Only Ahmad *et al*. used HeLa cells as the central cell line in their investigation – they showed the ability of CDDO-Me to interact directly with JAK1 and STAT3 and inhibit IL6-induced activation of the STAT3 pathway in these cells (1 μM (2 h, 6 h)) [116].

This work aims to reveal the mechanism of action of cyano enone-bearing triterpenoid SM in KB-3-1 human CC cells by the analysis of transcriptome changes induced by the compound. We concentrated our attention on gene expression profiles changed by SM during the first 10 h of treatment before the triggering of overall apoptosis and tried to restore key processes, upstream of cell death, switched on by the triterpenoid in these cells by using an *in silico* approach.

We showed that SM treatment lead to statistically significant modulation of the expression of a total of 1245 genes (fold change > 1.5, *p* < 0.05) (Figure 1B). The response of KB-3-1 cells to SM develops within the first hour of treatment, where 56 DEGs are detected (Figures 1B, 1D, 2), and reaches a conditional intermediate peak at 4 h – at this time point the central core associated with ER stress appears in the revealed functional interactome (Figure 2, 4h); later, this core retains its central position and is surrounded by new functional terms associated with ER homeostasis (Figure 3). According to our results, the strongest changes in intracellular signaling develop at 6 h of SM treatment – enrichment of a high number of functional terms along with the supremacy of up-regulated DEGs in it are clearly observed at this time point (Figure 3, 6 h). After 10 h of SM treatment, the functional interactome undergoes further changes – here we show a significant shift of gene set enrichment to down-regulated DEGs that can be explained by the inhibition of multiple processes in cells by SM; only several functional groups are shown to be characterized by high prevalence of up-regulated DEGs at this time point (Figure 3, 10 h).

Analysis of early DEGs changing their expression at the 1–4 h time points, in ClueGO plugin or manually by using GeneCards database shows that SM is able to affect a wide range of intracellular processes. In addition to dysregulation of the ER mentioned above, SM is found to negatively influence cholesterol metabolism, Ca^2+^ signaling, organization of the cytoskeleton and its related processes – endocytosis and intracellular transport – genes, involved in the regulation of mentioned pathways, are found as both unique and common DEGs in at least two time points during the analyzed period (1–4 h).

An increase of treatment time with SM to 6 h leads to the significant increase of the enrichment of cytoprotective functional groups associated with cell survival, including HIF-1 and IL-17 signaling, one carbon metabolism, and terms associated with compensation of nutrient failures, including response to starvation, transmembrane transport of amino acids and monosaccharide biosynthesis (Figure 3, 6 h). Although autophagy, detected at 6 h as a significantly changed pathway (Figure 3, 6h), could also play a cytoprotective role [43], we supposed that in the case of SM this process should be considered as cytodestructive. Previously, SM’s structural analog CDDO-Me was shown to induce autophagic cell death in different tumor cell lines (0.5-1 μM; K526/KBM5 leukemia (24 h) and Ec109/KYSE70 esophageal cancer cells (24 h)) [44, 123, 124], but additional studies in this field are required. The high enrichment of cytoprotective functional terms against the ER dysregulation background at the 6 h time point could indicate the triggering of a compensatory response of KB-3-1 cells to SM-induced stress. However, according to the revealed up-regulation of apoptosis-related genes and the corresponding appearance of a number of functional terms associated with cell death at the late phase of triterpenoid action (Figure 3, 10 h), detected cytoprotective mechanisms are insufficient to effectively resist SM-induced stress.

Considering the poor study of the mechanisms underlying the inhibitory effect of PTs on the growth of CC cell lines (Supplementary Table 1), our attention was also directed to the influence of SM on the expression of key genes, playing an important role in the vitality of this type of cells. Re-analysis of biopsy-based microarray data for CESC patients deposited in the GEO repository revealed 520 DEGs in tumor tissues as compared to their normal healthy counterparts, being common for three independent GEO datasets (Figure 7A), which can probably regulate malignant growth of CC cells (Figure 7B). We found that SM can modulate the expression of 57 of 520 CESC-related DEGs (Figure 7A, 7C); moreover, triterpenoid was shown to reverse the expression of the majority of revealed genes (Figure 7C). An example of a CESC-related key DEG, sensible for SM treatment, is *KLF4*, encoding Krüppel-like factor 4, the expression of which is significantly down-regulated in many types of tumors, including CESC [125]. It was found that KLF4 displays a suppressive effect on the growth of CC cells – overexpression of exogenous KLF in SiHa and C33A cells was shown to significantly reduce their proliferative ability [125]. Observed SM-induced up-regulation of KLF4 is in accordance with published data: it was shown that CDODA-Me and cyano enone-bearing derivative of betulinic acid induced KLF4 expression in colon cancer cells (1-5 μM (SW480, HT-29, HCT-15 cells (12 h)) and 5-10 μM (SW80, HT-29 cells (24 h)), respectively) [53, 126]. Along with *KLF4*, other CESC-associated DEGs *DUSP1*, *EGR1* and *FOS* were found to be sensitive to treatment with cyano enone-bearing triterpenoids: up-regulation of these genes and their proteins were detected in HUVEC and VC1 cells treated by CDDO-Im (0.05-0.2 μM (0.5–6 h)) [66, 127, 128].

In order to identify probable master genes among revealed CESC-associated DEGs, we analyzed them in the TCGA database and identified 4 genes (*EFNA1*, *TFRC*, *HIST1H2BK*, *ISG20*) out of 57 responding to SM treatment, the expression of which was associated with poor clinical outcomes in CESC patients (Figure 7D). The revealed genes *EFNA1* and *TFRC* encode ephrin A1 and the transferrin receptor, which play an important role in tumor development through inducing tumor angiogenesis and iron uptake, respectively [129, 130]. Previously, EFNA1 and TFRC have been proposed as prognostic factors in CESC [129, 131] and were found to be sensible to natural compounds – it was shown, that (-)-epigallocatechingallate and curcumin treatment, similar to SM, significantly decrease expression of EFNA1 and TFRC in tumor cells, respectively [132, 133]. *HIST1H2BK* encodes a core component of the nucleosome, which is probably important for tumor growth – previously, *HIST1H2BK* was revealed as a member of the most significant module and a hub node in gene regulatory networks in cervical and invasive ductal carcinomas, respectively [134, 135]. *ISG20*, relating to interferon-induced genes, encodes RNA exonuclease that displays antiviral activity. Although specific functions of ISG20 in tumor cells are still largely unknown, its importance for CC growth can be explained by the aetiological role of human papillomavirus in this cancer type. The involvement of *ISG20* in the CC-specific cellular response to SM was corroborated by the presence of this gene in the one of the major modules in the CC-associated gene regulatory network reconstructed by Mine *et al* [136]. To the best of our knowledge, SM is not the only stimulator of *ISG20* in the triterpenoid family – Watanabe *et al*. showed that the lanostane-type triterpenoid toosendanin increased *ISG20* expression in human hepatoma cells [137]. In view of the links between the expression levels of *EFNA1*, *TFRC*, *HIST1H2BK* and *ISG20* with the severity of CESC growth, protein products of these genes can act as CC-specific master regulators, probably playing an important role in the process of SM-induced death of KB-3-1 cells.

Following analysis of transcriptome data by several independent approaches (analysis of co-expressed DEGs, promotor analysis, PPI network reconstruction) it was confirmed that ER dysregulation and subsequent activation of ER stress are central events in processes triggered by SM in KB-3-1 cells. This is evidenced by (a) the functional annotation of the most significant STEM cluster 1 (Figure 4B), (b) identification of key ER stress markers ATF4 and DDIT3 not only as key nodes in the PPI network (Figure 4C), but also as transcription factors, probably being able to regulate the expression of DEGs, included in STEM cluster 1 (Figure 4D) and (c) detection of a range of ER stress-related functional groups during pathway analysis of significant modules in the PPI network (Figure 6C, green outlines).

Complex bioinformatic analysis also revealed several other pathways, dysregulated by SM and identified simultaneously by diverse methods, which can probably play a crucial role in the response of tumor cells to SM treatment. Such pathways included NF-kB survival signaling that was detected by the functional annotation of DEGs, revealed at 2 h of SM treatment (Figure 2, 2h), STEM cluster 1 (Figure 4B) or module 4 in the PPI network (Figure 6C). Moreover, the p50 subunit of NF-kB (NFKB1) was identified as a master regulator by promoter analysis of DEGs from STEM cluster 1 (Figure 4D). Observed high enrichment of NF-kB signaling pathway was probably explained by the triggering of a compensatory mechanism into KB-3-1 cells in response to SM and can be linked with SM-induced ER stress. Besides this, SM was found to be able to affect a range of intranuclear pathways, including DNA replication, nucleotide excision reparation and mRNA processing, which were identified by functional analysis of STEM cluster 2 (Figure 5B) and major modules in the PPI network (Figure 6C). Due to the fact that these functional terms consisted of down-regulated DEGs (Figure 6A), we supposed that SM can inhibit these vital cellular processes.

Comparison of obtained results revealed that SM can control actin cytoskeleton remodeling. This is evidenced by the identification of DEGs involved in the regulation of this process (*ACTB*, *ARPC4*, *CTTN*, *RAC1*, *VCL*) (Figure 5B, shigellosis), identification of ACTB in the Top-10 key nodes in STEM cluster 2 (Figure 5C) and detection of functional terms associated with actin cytoskeleton reorganization in module 2 in the PPI network – membrane trafficking and endocytosis (Figure 6C). The observed high enrichment of actin cytoskeleton-related terms in response to SM is in accordance with published data. To and colleagues found that CDDO-Me inhibited branched actin polymerization in Rat2 fibroblasts and, moreover, can directly bind to ACTB and actin-related proteins (1 μM; Rat2 fibroblasts (2 h)) [78].

**Figure 9:**
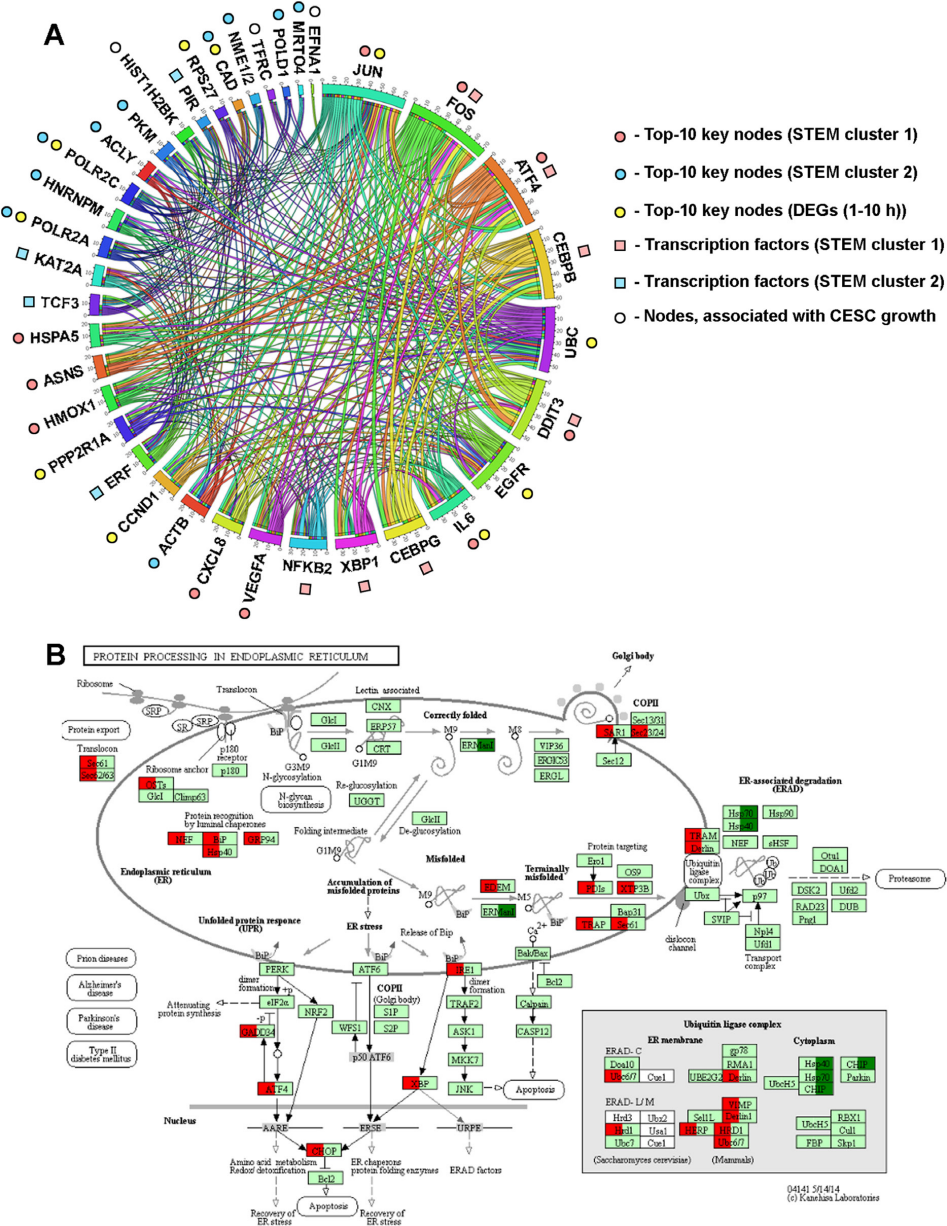
Dysregulation of ER homeostasis is a central event triggered by SM in KB-3-1 cells. (**A**) Interactome of MRs likely controlling the response of KB-3-1 cells to SM treatment. The regulatory network reconstructed with MRs was identified by different approaches during the study. Edges were retrieved by Cytoscape from STRING (confidence score > 0.7), UniProt, InnateDB, MINT and Mentha databases and regulatory interactions, revealed by promotor analysis of DEGs by using TRANSFAC, Jaspar, Encode, Swissregulon and Homer databases. The revealed interactome was visualized via Circos (http://mkweb.bcgsc.ca/tableviewer/). (**B**) The effect of SM treatment (10 h) on the expression of genes involved in the regulation of ER stress. Red and green colors show up- and down-regulated genes (fold change > 1.5, *p* < 0.05), respectively. Expression data was overlaid on the KEGG pathway 04141 via KeggExp (http://www.fgvis.com/expressvis/KeggExp/).

As a result of the performed integrated analysis we identified a range of master regulators, which probably control the response of KB-3-1 cells to SM – key nodes in PPI networks (Figures 4C, 5C, 6A), transcription factors (Figures 4D, 5D) and proteins, encoded by DEGs, expression of which is associated with high tumor growth in CESC patients (Figures 6D, 7D). Due to the high diversity of the revealed master regulators, we wondered which molecules from the revealed list were the most crucial for the regulation of SM-induced cellular stress. In order to understand this, we reconstructed the gene regulatory network for the revealed master regulators (Figure 9), and to increase the credibility of the regulatory relationships between nodes, interactions were retrieved from five databases (STRING, UniProt, InnateDB, MINT, Mentha) at once and additionally reinforced by regulatory data “TF - dependent gene”, obtained previously by the iRegulon tool (Figures 4D, 5D). As shown in Figure 9, all revealed master regulators, except ISG20, were highly interconnected with each other. It was found that the most nodal molecules in the network were JUN and FOS, the major subunits of AP-1 transcription factor, that can indicate the probable important role of AP-1 in response of KB-3-1 cells to SM treatment. Revealed SM-induced up-regulation of both JUN and FOS (Figures 4C, 4D) agreed well with published data. Previously, CDDO-Me and CDDO-Im were shown to increase the expression of JUN (1 μM; H157 cells (2–8 h)) [138] and FOS (0.2 μM (6 h)) [66] in human lung cancer cells and HUVEC, respectively. However, as far as we know, the specific role of AP-1 in cellular stress, induced by cyano enone-bearing triterpenoids, is still unknown. We speculate that the observed enrichment of AP-1 subunits as master regulators (Figure 9) can be explained by the ER-targeted effect of SM: it is known that AP-1 is activated during ER stress [21] and, moreover, JUN can be a key transcriptional regulator of UPR in tumor cells [139]. Detailed analysis of the interconnection between SM-induced AP-1 activation and ER stress is the subject of our further investigations. It should be noted that, along with AP-1 subunits, ER stress-related proteins (ATF4 [21], CEBPB [140], UBC [141], DDIT3 (or CHOP) [21]) were identified in the network as the most major master regulators (Figure 9A) that independently confirmed the important contribution of ER dysregulation in SM-induced cellular stress and subsequent cell death.

The ER is the organelle responsible for calcium storage, proper protein folding and lipid biosynthesis. Different stress inducers, including chemotherapeutic agents, can cause accumulation of unfolded proteins in the ER lumen, which triggers UPR to alleviate this stress and restore ER homeostasis by an increase of expression of ER-resident chaperones (BiP, GRP94, etc.), which facilitate *de novo* protein folding, attenuation of protein translation and the removing of misfolded proteins by ER-associated degradation (ERAD) machinery via ubiquitin-dependent proteolysis [142]. Strong or prolonged ER stress can induce apoptosis via CHOP (or DDIT3) and ATF4 overexpression [21, 142]. Our analysis clearly showed that SM can disturb protein processing in the ER with subsequent activation of UPR (Figure 9B). SM treatment was found to up-regulate genes, encoding key participants of this pathway, involving ER luminal chaperones (BiP (or HSPA5) (Figures 4C, 9), GRP94, etc.), proteins of the ERAD system (TRAM, Derlin, Sec61, etc.), ubiquitin ligase complex (VIMP, HERP, HRD1, etc.) and PERK/ATF4/CHOP and IRE1α/XBP1/CHOP signaling axes (Figures 4C, 4D, 8), which linked ER stress with subsequent activation of apoptosis (Figure 9B).

The revealed activation of ER stress by SM agreed well with published data. It was shown that PTs induced ER stress in many types of tumor cells, including cervical carcinoma cells [102, 143–145]. Moreover, triterpenoid-triggered ER stress is tightly associated with activation of apoptosis and cell death – suppression of ER stress by inhibitors (salubrinal, sodium phenylbutyrate, TUDCA) or siRNA techniques was found to effectively attenuate the pro-apoptotic or cytotoxic effects of PTs [45, 93, 145, 146]. Despite the large number of studies examining the ER stress-induced activity of PTs, the underlying mechanism of activation of ER stress by these compounds is still poorly understood. Rise of intracellular [Ca^2+^] or ROS, autophagy and MAP kinase were identified as probable triterpenoid-sensitive triggers of ER stress. It was shown that inhibition of these inducers by chemical inhibitors significantly suppressed ER stress caused by triterpenoids [45, 93, 147, 148]. In order to reveal a probable mechanism of induction of ER stress by SM, CMap analysis was carried out and showed significant similarity of transcriptome modulation by SM with two well-known ER stress inducers – thapsigargin and geldanamycin, which are inhibitors of ER resident proteins SERCA [91] and Grp94 [90], respectively. Subsequent molecular docking simulations of SM to SERCA and Grp94 revealed that the triterpenoid could snugly fit into the active sites of these proteins in positions very close to that of both inhibitors (Figures 8B, 8C, upper boxes). Moreover, we showed the ability of SM to occupy the binding pockets of other SERCA and Grp94 inhibitors – cyclopiazonic acid and methyl 2-[2-(2-benzylpyridin-3-yl) ethyl]-3-chloro-4,6-dihydroxybenzoate, respectively (Figures 8B, 8C, lower boxes). These data indicate that SM could target SERCA and Grp94 in multiple sites and, therefore, inhibit their activities; however, additional investigations in this field are needed. Results obtained with docking studies are in line with published data. It was shown previously that a range of triterpenoids could bind to SERCA and inhibit its pump activity [94–96]. Moreover, the increase of intracellular Ca^2+^ concentration induced by CDDO-Me (1.5 μM; MDA-MB 435/MDA-MB 231/MCF-7 breast cancer cells (1–6 h)) was found to be independent of ER resident Ca^2+^ release receptor RyR [93] and, therefore, could be explained by other mechanisms, such as the dysregulation of reverse pumping of Ca^2+^ from the cytoplasm to the ER, performed by SERCA.

The revealed ability of SM to interact with Grp94 (Figure 8C) is also correlated with previous reports. Recently, Qin and colleagues showed that CDDO-Me can directly bind to Hsp90 – a cytoplasmic paralog of Grp94, displaying high homology with it [90], and effectively inhibiting its activity (1.25-5 μM; HO8910/SKOV3 ovarian cancer cells (3-24 h)) [98]. Thus, the performed docking simulation showed that SM can trigger ER stress by two pathways – by dysregulation of calcium homeostasis and/or disturbances of protein folding, mediated by key ER luminal chaperone Grp94.

In conclusion, the performed bioinformatic analysis of SM-induced changes of the transcriptome in human cervical carcinoma KB-3-1 cells in the time period preceding overall apoptosis activation, clearly showed that SM perturbed a wide range of metabolic processes in tumor cells, among which ER stress can be considered as the central SM-induced intracellular event. SM, presumably binding to and inhibiting the activity of SERCA and Grp94, dysregulated proper protein folding, leading to the activation of compensatory mechanisms. However, prolonged SM-induced ER stress overbalanced the cytoprotective resources of the cells, which caused the activation of apoptosis. This study also showed the ability of SM to modulate the expression of the key genes involved in the regulation of the high proliferative rate of CESC. The reconstruction of the proposed mechanism of SM action based on transcriptomic data revealed several very important molecular events triggered by the triterpenoid in CC cells, nevertheless further investigations are required to confirm these results experimentally. Altogether, our findings suggest a probable novel mechanism of action of cyano enone-bearing triterpenoids in CC cells and provide a basis for the better understanding of the intracellular processes in tumor cells switched on in response to triterpenoids.

## MATERIALS AND METHODS

### Chemicals and reagents

The chemical synthesis of soloxolone methyl (SM) has been described before [11]. This compound has been fully characterized by chemical analysis and nuclear magnetic resonance. SM was dissolved in DMSO (10 mM), and stock solutions were stored at –20° C prior to usage.

### Cell culture and treatment

Human cervical carcinoma KB-3-1 cells were obtained from the Russian Cell Culture Collection (St. Petersburg, Russia) and were cultured in Dulbecco’s modified Eagle’s medium (DMEM) supplemented with 10% (v/v) heat-inactivated fetal bovine serum and antibiotic-antimycotic solution (100 U/ml penicillin, 100 μg/ml streptomycin and 0.25 μg/ml amphotericin). Cells were incubated at 37° C in a humidified atmosphere containing 5% CO_2_.

### Cell viability analysis

KB-3-1 cells were seeded in 96-well plates at 7 × 10^3^ cells/well. The plates were incubated at 37° C in 5% CO_2_ for 24 h. The medium was replaced with fresh medium containing SM at 0.2 – 2 μM and the cells were further incubated for 24 h. Aliquots of MTT solution (Sigma-Aldrich, USA) (10 μl, 5 mg/ml) were added to each well, and the incubation was continued for an additional 3 h. The dark blue crystals of formazan forming within healthy cells were solubilized with DMSO and the absorbance was measured in a Multiscan RC plate reader (Thermo LabSystems, Finland) at a test and reference wavelengths of 570 nm and 620 nm, respectively. IC_50_ values were determined as the compound concentration required to decrease the A_570_ to 50% of the control value (non-treated cells) and were extrapolated from dose-response curves.

### Cell treatment and microarray analysis

Subconfluent cells were seeded the day before the treatment. The cells were treated by SM (1 μM) for 1, 2, 3, 4, 6, or 10 h, each treatment was prepared in duplicate. The cells treated by 0.1% DMSO were used as a control. After treatments, cells were harvested in TRIzol Reagent (Ambion, USA), frozen at –70° C and shipped to JSC Genoanalytica (Moscow, Russia), where cDNA microarray hybridization experiments were performed on the Illumina HumanHT-12 v4 Expression BeadChip (Illumina, USA). The raw microarray data obtained were further normalized and the fold changes between the mean expression values of the genes in SM-treated cells in comparison with the control DMSO-treated cells, the *p*-value and the adjusted *p*-value were calculated by using the geneXplain 2.3 v.3 platform (geneXplain GmbH, Germany). Intersections of differentially expressed genes (DEGs) in the early time period (1–4 h) and CESC-associated DEGs from different datasets were carried out by the Venny v.2.0 tool (http://bioinfogp.cnb.csic.es/tools/venny/). The heatmap of gene expression was generated by the ClusterMaker2 v.1.3.1 plugin in Cytoscape.

### Quantitative reverse-transcription PCR (qRT-PCR)

KB-3-1 cells were treated by SM at 1 μM for 10 h, harvested and the total RNA was extracted in Trizol (Ambion, USA) according to the manufacturer’s protocol. The first-strand of cDNA was synthesized using 1.5 μg total RNA and M-MuLV-RH Reverse Transcription kit (Biolabmix, Russia). PCR was performed in triplicate using a BioMaster HS-qPCR SYBR Blue (2×) (Biolabmix. Russia) in a final reaction volume of 20 μl, containing 5 μl of cDNA template, with gene specific primer sets using an iQ5 Cycler (Bio-Rad, USA). The housekeeping gene *GAPDH* was used as a reference gene. The following primers were used in our study: *Bri3bp*, forward 5′-AGGCTGACTGAGAGATTTGT-3′ and reverse 5′-AAATACTGGGACAGGTTGGA-3′; *Ddit3*, forward 5′-CGACAGAGCCAAAATCAGAG-3′ and reverse 5′-TCAGGTGTGGTGATGTATGA-3′; *Fasn*, forward 5′-CAAGCTGAAGGACCTGTCTAG-3′ and reverse 5′-CGGAGTGAATCTGGGTTGATG-3′; *Gdf15* forward 5′-AAGATTCGAACACCGACCTC-3′ and reverse 5′-CCCGAGAGATACGCAGG-3′; *Herpud1* forward 5′-GATTGGACCTATTCAGCAGC-3′ and reverse 5′-GCCTCGGTCTAAATGGAAAC-3′; *Hmox1* forward 5′-AGAATGCTGAGTTCATGAGGA-3′ and reverse 5′-CATAGATGTGGTACAGGGAGG-3′; *Igfbp1*, forward 5′-CACAGGAGACATCAGGAGAAG-3′ and reverse 5′-GATCCTCTTCCCATTCCAAGG-3′; *Osgin1* forward 5′-CATGGTGATCCTGAGCCAAG-3′ and reverse 5′-ACGTAGTCCCTGTAGTAGTGG-3′; *Gapdh*, forward 5′-GTGAAGGTCGGAGTCAAC-3′ and reverse 5′-TGGAATTTGCCATGGGTG-3′. PCR specificity was controlled using melting curves. Calculation of relative gene expression (normalized to *GAPDH*) was performed according to the ΔΔC_T_ method.

### Functional enrichment analysis

To decipher the biological processes and pathways modulated by SM treatment, functional enrichment analysis of DEGs were performed using ClueGO v.2.5.1 [17] or CluePedia v.1.5.1 [149] plugins in Cytoscape. DEGs were mapped on the latest update of Gene Ontology (biological processes), Kyoto Encyclopedia of Genes and Genomes (KEGG), REACTOME and Wikipathways (released May 2018 (early and late DEGs), June 2018 (STEM clusters, PPI network) and August 2018 (CESC-associated DEGs)). The GO tree interval ranged from 3 to 8 with the minimum number of genes per cluster set to two (early and late DEGs), three (STEM clusters, PPI network) or four (CESC-associated DEGs). Term enrichment was tested with a two-sided hypergeometric test that was corrected by the Bonferroni method. Only terms with p ≤ 0.05 were included in the analysis. Functional grouping and linking of the enriched terms were performed with kappa statistics (kappa score 0.4 (early and late DEGs, STEM clusters, PPI network) or 0.7 (CESC-associated DEGs)). Functional annotation of early DEGs (1–4 h) was also performed manually using data deposited in the GeneCards database [150].

### Time course analysis

Identification of co-expressed genes was performed by the STEM clustering method [61] using the Short Time-series Expression Miner (STEM) v. 1.3.11 with the following settings: maximum number of model profiles = 50, maximum unit change in model profiles between time points = 2, number of permutations per gene = 50. Significant expression profiles were identified with *p* < 0.05 after Bonferroni correction.

### PPI network reconstruction

The protein-protein interactions (PPIs) were predicted based on data deposited in the STRING (Search Tool for the Retrieval of Interacting Genes) database [151] with a confidence score > 0.7. The protein pairs collection included functional relationships of proteins from five sources: genomic context prediction, co-expression, high-throughput lab experiment, automated text mining and known PPI from other databases. Reconstructed PPI networks were visualized by Cytoscape v. 3.6.1. To reveal hub proteins, characterized by the highest interconnection with neighbors in the PPI network, node degree scores were calculated using the NetworkAnalyzer plugin [152]. To detect densely connected regions in the reconstructed PPI network, a graph theoretic clustering algorithm was employed using the MCODE v. 1.5.1 tool [153] with degree cutoff = 2, node score cutoff = 0.2, k-score = 2, maximal depth = 100. PPI modules with a score > 5 were visualized by Cytoscape and functionally annotated using the ClueGO plugin.

In the case of the gene regulatory network, reconstructed from revealed probable master regulators (MRs), the interactive relationships among MRs were re-established based on data deposited in STRING (confidence score > 0.7), UniProt, InnateDB, MINT and Mentha databases (released February 2019) and regulatory data “transcription factor – target gene”, obtained by us previously, and visualized via Circos (http://mkweb.bcgsc.ca/tableviewer/) [154]. The mapping of expression data in KEGG pathways was carried out by using KeggExp (http://www.fgvis.com/expressvis/KeggExp/) [155].

### Prediction of transcription factors for STEM cluster genes

To identify transcription factors (TFs) likely involved in the regulation of expression of DEGs, composing STEM clusters, the iRegulon v. 1.3 [67] and geneXplain tools were used. The TF – target gene interactions were collected from the TRANSFAC [156], Jaspar, Encode, Swissregulon, Home and HOCOMOCO [71] databases. The set of DEGs was submitted to iRegulon and analyzed using the following options: motif collection = 10K (9713 PWMs), track collection = 1120 ChIP-seq tracks, putative regulatory region and motif ranking database = 10kb centered around TSS, minimum identity between orthologous genes = 0.05, maximum FDR on motif similarity = 0.001. Calculated Normalized Enrichment Score (NES) indicates the reliability of the results, and TFs, displaying regulatory associations with NES > 3, were selected for further analysis. In order to identify the potential regulatory relationship, the Pearson correlation coefficient was calculated for all pair-wise comparisons of gene expression profiles between selected TFs and DEGs [68]. The regulationship with r > 0.9 was considered as significant.

### Survival analysis of hub genes

To reveal the probable involvement of identified nodal DEGs in cervical carcinoma progression, survival analysis of DEGs was carried out using TCGA clinical data for patients with cervical squamous cell carcinoma and endocervical adenocarcinoma (CESC). The multivariative cox regressions and Kaplan-Meier analysis for CESC were performed using the OncoLnc tool (http://www.oncolnc.org/).

### GEO dataset processing

The gene expression profiles of GSE7410 (27 CESC tissue samples, 7 non-cancerous samples) GSE7803 (21 CESC tissue samples, 10 non-cancerous samples) and GSE63514 (28 CESC tissue samples, 24 non-cancerous samples) were obtained from the Gene Expression Omnibus (GEO, https://www.ncbi.nlm.nih.gov/geo) database. The identification of DEGs between CESC and healthy cervix tissue samples was carried out by GEO2R (https://www.ncbi.nlm.nih.gov/geo/geo2r/), an interactive web tool that allows users to compare two or more datasets in a GEO series in order to identify DEGs across experimental conditions [157]. The adjusted *p*-values were applied to correct the false positive results by the default Benjamini-Hochberg false discovery rate method. The adj. *p* < 0.05 and |Fold Change| > 1.5 were considered as the cutoff values.

### CMap analysis

To identify drugs with known intracellular targets, displaying similar effects on the transcriptome with SM, Connectivity Map analysis was conducted through the ToppGene Suite (https://toppgene.cchmc.org/) [158]. DEGs (SM-treated vs non-treated cells, fold change > 1.5, *p* < 0.05) identified at the 10 h time point were analyzed. Revealed candidate drugs were ranked by *p*-value and the Top-10 drugs were listed.

### Molecular docking

Docking of SM with SERCA and Grp94 was performed using Autodock Vina [159]. The three-dimensional structures of rabbit SERCA co-crystalized with thapsigargin and cyclopiazonic acid (PDB ID: 2AGV and 2OA0, respectively) and canine Grp94 co-crystalized with geldanamycin and methyl 2-[2-(2-benzylpyridin-3-yl) ethyl]-3-chloro-4,6-dihydroxybenzoate (PBD ID: 2EXL and 6ASQ, respectively) were obtained from the protein data bank (https://www.rcsb.org). Further, the extraction of co-crystalled ligands from the PDB files of proteins, addition of polar hydrogens and Gasteiger charges into protein structures were performed by the AutoDockTools v.1.5.7. The 2D structure of SM was converted to 3D and its geometry was optimized with the universal force field (UFF) using Mavin Sketch v.5.12 and Avogadro v. 1.2.0, respectively. All rotatable bonds within the ligand were allowed to rotate freely. The docking grid box sizes were set to 36 × 44 × 60 Å, 44 × 34 × 34 Å, 36 × 30 × 38 Å and 30 × 30 × 36 Å for 2AGV, 2OA0, 2EXL and 6ASQ, respectively, centered on the positions of the ligands ((–4.53)×(–23.105)×8.47 Å, 24.496×(–14.36) ×13.574 Å, 18.111 × 2.972×(–5.889) Å and (–53.674) ×164.902× (–53.456) Å for 2AGV, 2OA0, 2EXL and 6ASQ, respectively). Finally, the conformations with the most favorable free energy of binding were selected to analyze the interaction between SM and proteins. The results were imported and analyzed using Discovery Studio Visualizer v.17.2.0. The 2D plots of the protein-ligand interactions were analyzed using LigPlot+ v.1.4.5.

## SUPPLEMENTARY MATERIALS



## Abbreviations

AP-1: activator protein 1
CESC: cervical squamous cell carcinoma and endocervical adenocarcinoma
CMap: Connectivity Map
CN group: cyano group
CPA: cyclopiazonic acid
DEG: differentially expressed gene
ER: endoplasmic reticulum
ERAD: endoplasmic reticulum-associated protein degradation
GEO: The Gene Expression Omnibus
HIF-1: hypoxia inducible factor 1
HO-1: heme oxygenase 1
HUVEC: human umbilical vein endothelial cells
IC_50_: compound concentration resulting in 50% survival of cell
IL: interleukin
KEGG: Kyoto Encyclopedia of Genes and Genomes
NF-kB: nuclear factor κB
NFKB1: p50 subunit of NK-kB
PPARγ: peroxisome proliferator activated receptor g
PPI: protein-protein interaction network
PTs: pentacyclic triterpenoids
ROS: reactive oxygen species
RTK: receptor tyrosine kinase
SERCA: sarcoplasmic/endoplasmic reticulum Ca^2+^ ATPase
SM: soloxolone methyl
STEM: the Short Time-series Expression Miner
STRING: search tool for the retrieval of interacting genes/proteins
TCGA: The Cancer Genome Atlas
TF: transcription factor
tRNA: transfer RNA
UPR: unfolded protein response
VEGF: vascular endothelial growth factor
VEGFR: vascular endothelial growth factor receptor 2
