# Supplementary material for: Deep insights into the response of human cervical carcinoma cells to a new cyano enone-bearing triterpenoid soloxolone methyl: a transcriptome analysis
Source: Oncotarget. 2019 Sep 3;10(51):5267–97. doi: 10.18632/oncotarget.27085 (PMC6731101; doi:10.18632/oncotarget.27085)
Supplement: Supplementary file 1 [file oncotarget-10-5267-s001.pdf]

Deep insights into the response of human cervical carcinoma cells to a new cyano enone-bearing triterpenoid soloxolone methyl: a transcriptome analysis

SUPPLEMENTARY MATERIALS

REFERENCES

1. Lee CS, Kim YJ, Lee MS, Han ES, Lee SJ. 18β-Glycyrrhetinic acid induces apoptotic cell death in SiHa cells and exhibits a synergistic effect against antibiotic anti-cancer drug toxicity. Life Sci. 2008; 83:481–489. https://doi.org/10.1016/j.lfs.2008.07.014. [PubMed]
2. Xu Y, Ching YP, Zhou Y, Chiu JF, Chen F, He QY. Multiple pathways were involved in tubeimoside-1-induced cytotoxicity of HeLa cells. J Proteomics. 2011; 75:491–501. https://doi.org/10.1016/j.jprot.2011.08.014. [PubMed]
3. Sun HX, Zheng QF, Tu J. Induction of apoptosis in HeLa cells by 3β-hydroxy-12-oleanen-27-oic acid from the rhizomes of Astilbe chinensis. Bioorg Med Chem. 2006; 14:1189–1198. https://doi.org/10.1016/j.bmc.2005.09.043. [PubMed]
4. Wang R, Li Y, Huai XD, Zheng QX, Wang W, Li HJ, Huai QY. Design and preparation of derivatives of oleanolic and glycyrrhetinic acids with cytotoxic properties. Drug Des Devel Ther. 2018; 12:1321–1336. https://doi.org/10.2147/DDDT.S166051. [PubMed]
5. Wang Y, Porter WW, Suh N, Honda T, Gribble GW, Leesnitzer LM, Plunket KD, Mangelsdorf DJ, Blanchard SG, Willson TM, Sporn MB. A Synthetic Triterpenoid, 2-Cyano-3,12-dioxooleana-1,9-dien-28-oic Acid (CDDO), Is a Ligand for the Peroxisome Proliferator-Activated Receptor. Mol Endocrinol. 2000; 14:1550–1556. https://doi.org/10.1210/mend.14.10.0545. [PubMed]
6. Bernstein SH, Venkatesh S, Li M, Lee J, Lu B, Hilchey SP, Morse KM, Metcalfe HM, Skalska J, Andreeff M, Brookes PS, Suzuki CK. The mitochondrial ATP-dependent Lon protease: a novel target in lymphoma death mediated by the synthetic triterpenoid CDDO and its derivatives. Blood. 2012; 119:3321–9. https://doi.org/10.1182/blood-2011-02-340075. [PubMed]. Erratum in: Blood. 2014 Aug 28; 124(9):1539. https://doi.org/10.1182/blood-2014-07-587428.
7. Ahmad R, Raina D, Meyer C, Kufe D. Triterpenoid CDDO-methyl ester inhibits the Janus-activated kinase-1 (JAK1)®signal transducer and activator of transcription-3 (STAT3) pathway by direct inhibition of JAK1 and STAT3. Cancer Res. 2008; 68:2920–2926. https://doi.org/10.1158/0008-5472.CAN-07-3036. [PubMed]
8. Benosman S, Ravanan P, Correa RG, Hou YC, Yu M, Gulen MF, Li X, Thomas J, Cuddy M, Matsuzawa Y, Sano R, Diaz P, Matsuzawa S, Reed JC. Interleukin-1 Receptor-Associated Kinase-2 (IRAK2) Is a Critical Mediator of Endoplasmic Reticulum (ER) Stress Signaling. PLoS One. 2013; 8:e64256. https://doi.org/10.1371/journal.pone.0064256. [PubMed]
9. Daroczi B, Kari G, Ren Q, Dicker AP, Rodeck U. Nuclear factor κB inhibitors alleviate and the proteasome inhibitor PS-341 exacerbates radiation toxicity in zebrafish embryos. Mol Cancer Ther. 2009; 8:2625–2634. https://doi.org/10.1158/1535-7163.MCT-09-0198. [PubMed]
10. Probst BL, Trevino I, McCauley L, Bumeister R, Dulubova I, Wigley WC, Ferguson DA. RTA 408, a novel synthetic triterpenoid with broad anticancer and anti-inflammatory activity. PLoS One. 2015; 10:e0122942. https://doi.org/10.1371/journal.pone.0122942. [PubMed]
11. Logashenko EB, Salomatina OV, Markov AV, Korchagina DV, Salakhutdinov NF, Tolstikov GA, Vlassov VV, Zenkova MA. Synthesis and Pro-Apoptotic Activity of Novel Glycyrrhetinic Acid Derivatives. ChemBioChem. 2011; 12:784–794. https://doi.org/10.1002/cbic.201000618. [PubMed]
12. Ravanan P, Singh SK, Rao GS, Kondaiah P. Growth inhibitory, apoptotic and anti-inflammatory activities displayed by a novel modified triterpenoid, cyano enone of methyl boswellates. J Biosci. 2011; 36:297–307. https://doi.org/10.1007/s12038-011-9056-7. [PubMed]
13. Kaur R, Khan S, Chib R, Kaur T, Sharma PR, Singh J, Shah BA, Taneja SC. A comparative study of proapoptotic potential of cyano analogues of boswellic acid and 11-keto-boswellic acid. Eur J Med Chem. 2011; 46:1356–1366. https://doi.org/10.1016/j.ejmech.2011.01.061. [PubMed]
14. Yim EK, Lee MJ, Lee KH, Um SJ, Park JS. Antiproliferative and antiviral mechanisms of ursolic acid and dexamethasone in cervical carcinoma cell lines. Int J Gynecol Cancer. 2006; 16:2023–2031. https://doi.org/10.1111/j.1525-1438.2006.00726.x. [PubMed]
15. Li Y, Lu X, Qi H, Li X, Xiao X, Gao J. Ursolic Acid Induces Apoptosis Through Mitochondrial Intrinsic Pathway and Suppression of ERK1/2 MAPK in HeLa Cells. J Pharmacol Sci. 2014; 125:202–210. https://doi.org/10.1254/jphs.14017FP. [PubMed]

16. Li L, Hou Y, Yu J, Lu Y, Chang L, Jiang M, Wu X. Synergism of ursolic acid and cisplatin promotes apoptosis and enhances growth inhibition of cervical cancer cells via suppressing NF-κB p65. Oncotarget. 2017; 8:97416–97427. https://doi.org/10.18632/oncotarget.22133. [PubMed]

17. Wang S, Meng X, Dong Y. Ursolic acid nanoparticles inhibit cervical cancer growth *in vitro* and *in vivo* via apoptosis induction. Int J Oncol. 2017; 50:1330–1340. https://doi.org/10.3892/ijo.2017.3890. [PubMed]

18. Xu T, Pang Q, Wang Y, Yan X. Betulinic acid induces apoptosis by regulating PI3K/Akt signaling and mitochondrial pathways in human cervical cancer cells. Int J Mol Med. 2017; 40:1669–1678. https://doi.org/10.3892/ijmm.2017.3163. [PubMed]

19. Xu T, Pang Q, Zhou D, Zhang A, Luo S, Wang Y, Yan X. Proteomic investigation into betulinic acid-induced apoptosis of human cervical cancer HeLa cells. PLoS One. 2014; 9:e105768. https://doi.org/10.1371/journal.pone.0105768. [PubMed]

20. Wang P, Li Q, Li K, Zhang X, Han Z, Wang J, Gao D, Li J. Betulinic acid exerts immunoregulation and anti-tumor effect on cervical carcinoma (U14) tumor-bearing mice. Pharmazie. 2012; 67:733–739. https://doi.org/10.1691/ph.2012.1822. [PubMed]

21. Prasad N, Sabarwal A, Yadav UCS, Singh RP. Lupeol induces S-phase arrest and mitochondria-mediated apoptosis in cervical cancer cells. J Biosci. 2018; 43:249–261. https://doi.org/10.1007/s12038-018-9743-8. [PubMed]

22. Ghosh S, Mukhopadhyay S, Sarkar M, Mandal A, Das V, Kumar A, Giri B. Biological evaluation of a halogenated triterpenoid, 2α-bromo-dihydrobelulonic acid as inhibitor of human topoisomerase IIα and HeLa cell proliferation. Chem Biol Interact. 2017; 268:68–76. https://doi.org/10.1016/j.cbi.2017.02.015. [PubMed]

23. Wong YH, Abdul Kadir H. Induction of mitochondria-mediated apoptosis in ca ski human cervical cancer cells triggered by mollic acid arabinoside isolated from Leea indica. Evid Based Complement Alternat Med. 2012; 2012:684740. https://doi.org/10.1155/2012/684740. [PubMed]

24. Iseli TJ, Turner N, Zeng XY, Cooney GJ, Kraegen EW, Yao S, Ye Y, James DE, Ye JM. Activation of AMPK by Bitter Melon Triterpenoids Involves CaMKKβ. PLoS One. 2013; 8:e62309. https://doi.org/10.1371/journal.pone.0062309. [PubMed]

25. Hata K, Mukaiyama T, Tsujimura N, Sato Y, Kosaka Y, Sakamoto K, Hori K. Differentiation-inducing activity of lupane triterpenes on a mouse melanoma cell line. Cytotechnology. 2006; 52:151–158. https://doi.org/10.1007/s10616-007-9069-0. [PubMed]

26. Zhang H, Wang Y, Zhu P, Liu J, Xu S, Yao H, Jiang J, Ye W, Wu X, Xu J. Design, synthesis and antitumor activity of triterpenoid pyrazine derivatives from 23-hydroxybetulinic acid. Eur J Med Chem. 2015; 97:235–244. https://doi.org/10.1016/j.ejmech.2015.04.057. [PubMed]

27. Byun JY, Kim MJ, Eum DY, Yoon CH, Seo WD, Park KH, Hyun JW, Lee YS, Lee JS, Yoon MY, Lee SJ. Reactive Oxygen Species-Dependent Activation of Bax and Poly(ADP-ribose) Polymerase-1 Is Required for Mitochondrial Cell Death Induced by Triterpenoid Pristimerin in Human Cervical Cancer Cells. Mol Pharmacol. 2009; 76:734–744. https://doi.org/10.1124/mol.109.056259. [PubMed]

28. Liu RM, Li YB, Zhong JJ. Cytotoxic and pro-apoptotic effects of novel ganoderic acid derivatives on human cervical cancer cells *in vitro*. Eur J Pharmacol. 2012; 681:23–33. https://doi.org/10.1016/j.ejphar.2012.02.007. [PubMed]

29. Li X, Bau T, Bao H. FPOA induces apoptosis in HeLa human cervical cancer cells through a caspase-mediated pathway. Oncol Lett. 2018; 15:8357–8362. https://doi.org/10.3892/ol.2018.8380. [PubMed]

30. Zhao LW, Zhong XH, Yang SY, Zhang YZ, Yang NJ. Inotodiol Inhabits Proliferation and Induces Apoptosis through Modulating Expression of cyclinE, p27, bcl-2, and bax in Human Cervical Cancer HeLa Cells. Asian Pac J Cancer Prev. 2014; 15:3195–3199. https://doi.org/10.7314/APJCP.2014.15.7.3195. [PubMed]

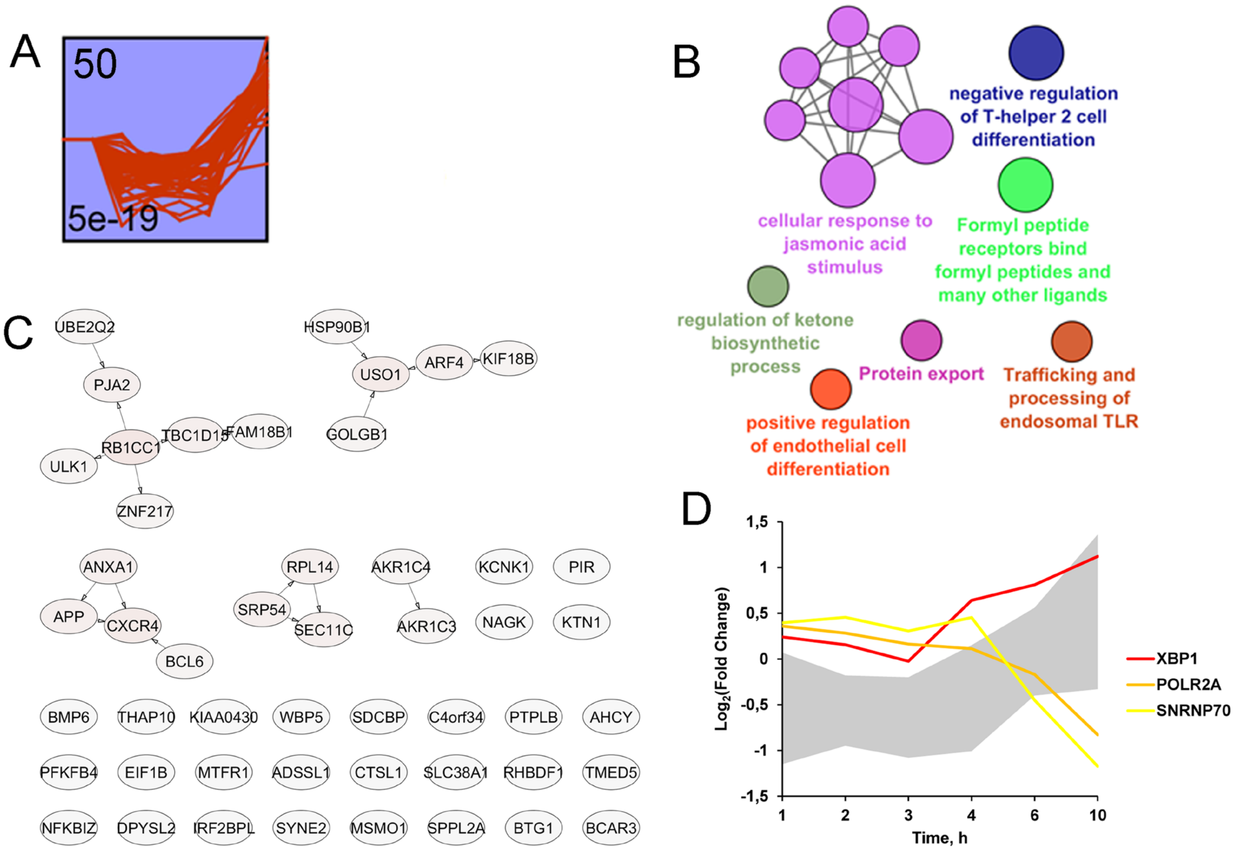

TF	NES	Target genes	r
XBP1	5.116	19	0.93
POLR2A	3.62	21	-0.96
SNRNP70	3.099	23	-0.98

Supplementary Figure 1: STEM cluster 3. (**A**) Expression profile of DEGs found in Cluster 3. The number in the top left corner represents the number of DEGs included in the cluster; the bottom left corner shows the p-value of the profile. X and Y-axes represent time points and relative gene expression levels, respectively. (**B**) The interaction network of significant terms enriched with DEGs from Cluster 3. Functional annotation was performed in ClueGO by using Gene Ontology (biological processes), KEGG, REACTOME and Wikipathways. The functionally grouped network is linked based on the kappa score of terms. The size and color of a node represents the term's significance and its attachment to revealed groups. Only pathways with $p < 0.05$ after Bonferroni step down correction for multiple testing were included in the networks. (**C**) The protein-protein interaction network reconstructed with DEGs from Cluster 3. The red gradient is proportional to the number of interactions (degree) of nodes with neighbors. (**D**) Potential transcription factors (TFs), regulating genes included in Cluster 3, were identified by iRegulon. TFs, characterized by normalized enrichment score (NES) > 3 and high correlation between TFs and target DEGs expression profiles ($|r| > 0.9$), are shown. The gray area and colored lines in the diagram represent the expression profile of DEGs and identified TFs, respectively.

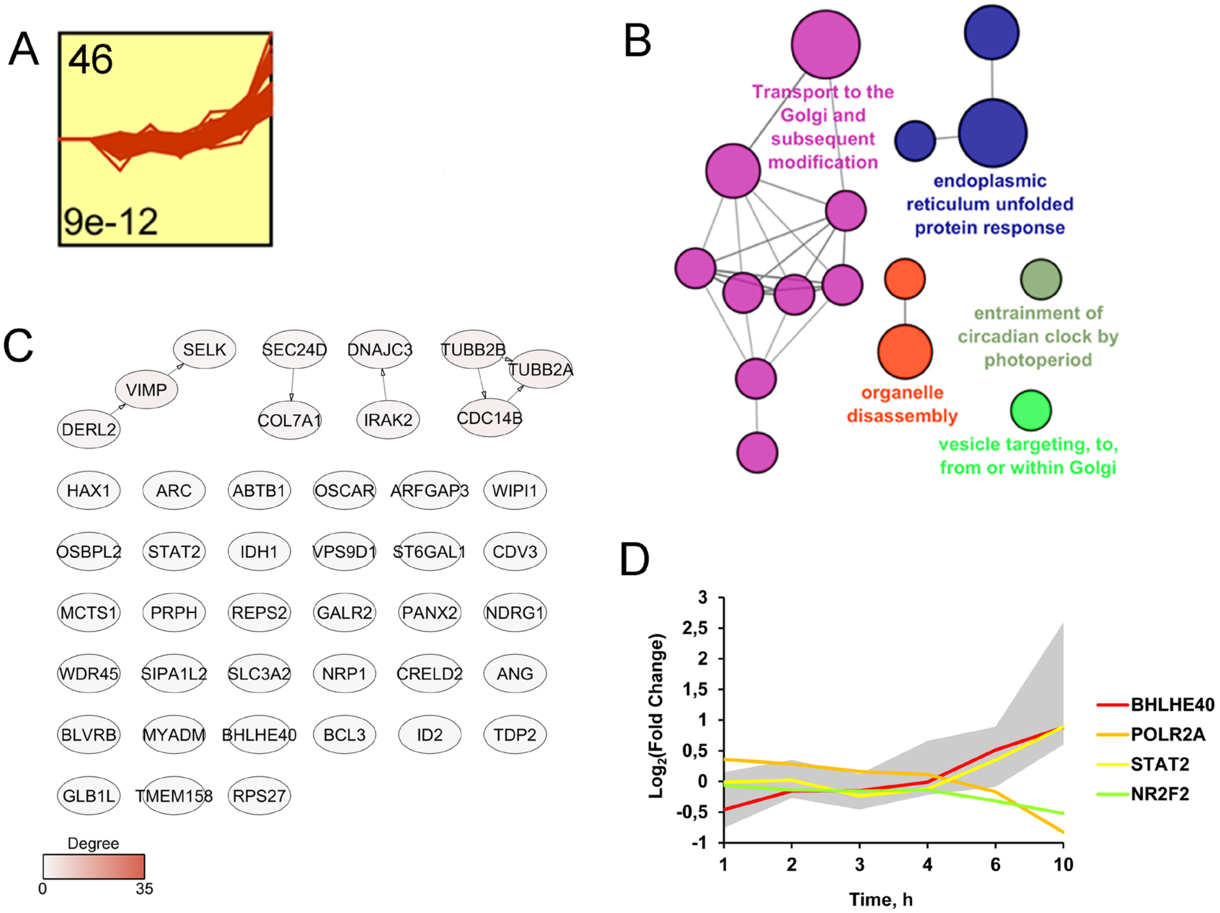

TF	NES	Target genes	r
BHLHE40	4.069	14	0.93
POLR2A	3.803	7	-0.96
STAT2	3.691	16	0.91
NR2F2	3.456	8	-0.95

Supplementary Figure 2: STEM cluster 4. (**A**) Expression profile of DEGs found in Cluster 4. The number in the top left corner represents the number of DEGs included in the cluster; the bottom left corner shows the p-value of the profile. X and Y-axes represent time points and relative gene expression levels, respectively. (**B**) The interaction network of significant terms enriched with DEGs from Cluster 4. Functional annotation was performed in ClueGO by using Gene Ontology (biological processes), KEGG, REACTOME and Wikipathways. The functionally grouped network is linked based on the kappa score of terms. The size and color of a node represents the term's significance and its attachment to revealed groups. Only pathways with $p < 0.05$ after Bonferroni step down correction for multiple testing were included in the networks. (**C**) The protein-protein interaction network reconstructed with DEGs from Cluster 3. The red gradient is proportional to the number of interactions (degree) of nodes with neighbors. (**D**) Potential transcription factors (TFs), regulating genes included in Cluster 4, were identified by iRegulon. TFs, characterized by normalized enrichment score (NES) > 3 and high correlation between TFs and target DEGs expression profiles ($|r| > 0.9$), are shown. The gray area and colored lines in the diagram represent the expression profile of DEGs and identified TFs, respectively.

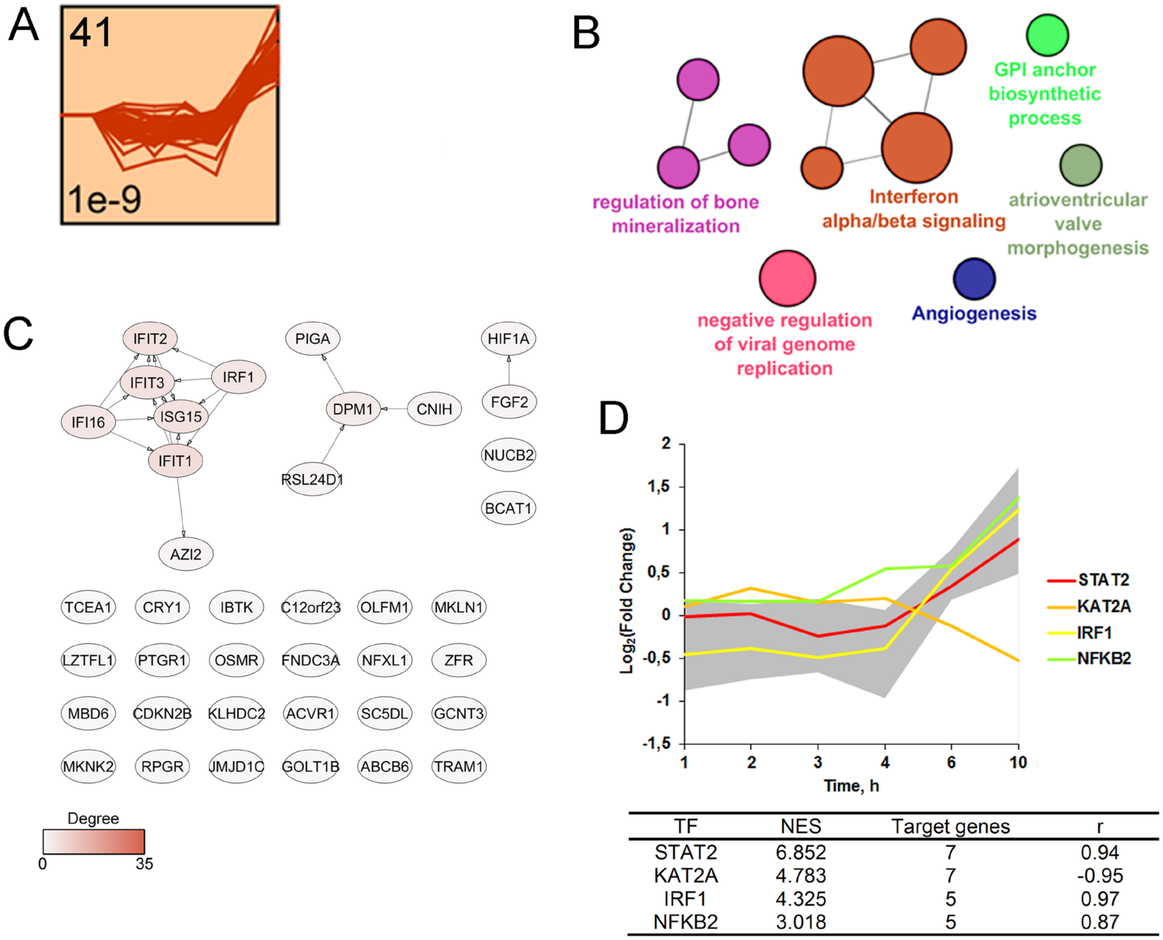

TF	NES	Target genes	r
STAT2	6.852	7	0.94
KAT2A	4.783	7	-0.95
IRF1	4.325	5	0.97
NFKB2	3.018	5	0.87

Supplementary Figure 3: STEM cluster 5. (**A**) Expression profile of DEGs found in Cluster 5. The number in the top left corner represents the number of DEGs included in the cluster; the bottom left corner shows the p-value of the profile. X and Y-axes represent time points and relative gene expression levels, respectively. (**B**) The interaction network of significant terms enriched with DEGs from Cluster 5. Functional annotation was performed in ClueGO by using Gene Ontology (biological processes), KEGG, REACTOME and Wikipathways. The functionally grouped network is linked based on the kappa score of terms. The size and color of a node represents the term's significance and its attachment to revealed groups. Only pathways with $p < 0.05$ after Bonferroni step down correction for multiple testing were included in the networks. (**C**) The protein-protein interaction network reconstructed with DEGs from Cluster 5. The red gradient is proportional to the number of interactions (degree) of nodes with neighbors. (**D**) Potential transcription factors (TFs), regulating genes included in Cluster 5, were identified by iRegulon. TFs, characterized by normalized enrichment score (NES) > 3 and high correlation between TFs and target DEGs expression profiles ($|r| > 0.9$), are shown. The gray area and colored lines in the diagram represent the expression profile of DEGs and identified TFs, respectively.

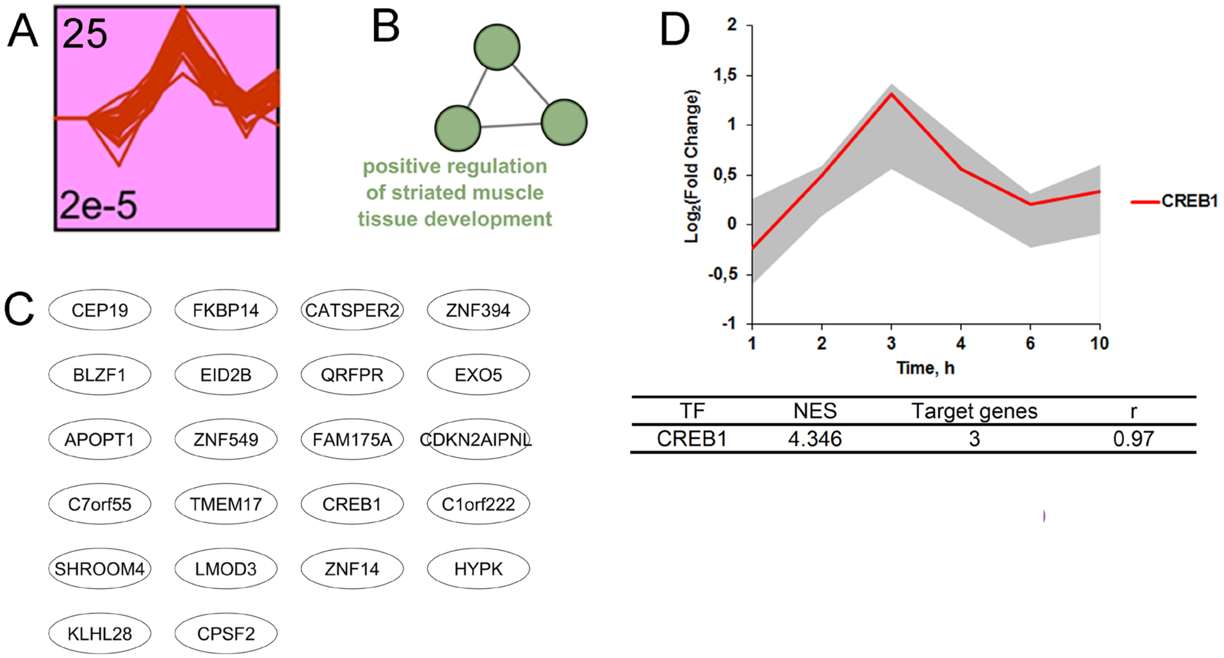

TF	NES	Target genes	r
CREB1	4.346	3	0.97

Supplementary Figure 4: STEM cluster 6. (**A**) Expression profile of DEGs found in Cluster 6. The number in the top left corner represents the number of DEGs included in the cluster; the bottom left corner shows the p-value of the profile. X and Y-axes represent time points and relative gene expression levels, respectively. (**B**) The interaction network of significant terms enriched with DEGs from Cluster 6. Functional annotation was performed in ClueGO by using Gene Ontology (biological processes), KEGG, REACTOME and Wikipathways. The functionally grouped network is linked based on the kappa score of terms. The size and color of a node represents the term's significance and its attachment to revealed groups. Only pathways with $p < 0.05$ after Bonferroni step down correction for multiple testing were included in the networks. (**C**) The protein-protein interaction network reconstructed with DEGs from Cluster 6. (**D**) Potential transcription factors (TFs), regulating genes included in Cluster 6, were identified by iRegulon. TFs, characterized by normalized enrichment score (NES) > 3 and high correlation between TFs and target DEGs expression profiles ($|r| > 0.9$), are shown. The gray area and colored line in the diagram represent the expression profile of DEGs and identified TF, respectively.

Supplementary Table 1: Effect of triterpenoids on cervical carcinoma cells[a]

Type	Compound	Cell line	Effect	Upstream cell death-associated target	Ref.
Oleanane	18β-glycyrrhetinic acid	SiHa	Cytotoxic (IC_{50}~37 μM), ↑nuclear damage, ↑Bax/Bcl-2, ↑cytochrome c release, ↑caspase-3 activity, ↓$\Delta\psi_M$[b], ↓ROS, ↓GSH, synergism with mitomycin c, doxorubicin	ROS (oxidant scavengers pretreatment → ↑GA-induced cell death)	[1]
	Tubeimoside-1	HeLa	Proteomics revealed enrichment of proteins (a) located in mitochondria involving in energy production, (b) located in ER as chaperones functioning for protein folding, (c) related to cytoskeleton function; ↑TUFM, ↓VDAC2, ↓Prx1, ↑keratin-10, ↑keratin-16, ↑annexin-1; ↑ROS, ↓pro-caspase-12, G_2/M arrest, ↑p-Chk2, ↓Cdc25C, ↑p- Cdc25C, ↓Cdc2, slight ↑p-Cdc2, ↓Cyclin B, ↑p21, ↑cytoplasmic Cdc25C, ↓α-tubulin	ATM kinase (caffeine pretreatment → ↓tubeimoside-1-induced G_2/M arrest) Ca^{2+} homeostasis (BAPTA-AM pretreatment → ↓tubeimoside-1-indeuced cell death)	[2]
	3β-hydroxy-12-oleanen-27-oic acid	HeLa	↓cell viability (IC_{50} = 6.49 ± 0.33 μg/ml), ↓total protein content, ↑nuclear condensation, ↑nuclear fragmentation, ↑membrane blebbing, ↑apoptotic bodies, ↑chromatin condensation, ↑DNA fragmentation, G_0/G_1 arrest, ↑subG1, ↑Bax/Bcl-2, ↓$\Delta\psi_M$	Caspase-3/7 (DEVD-CHO pretreatment → ↓triterpenoid-induced cell death)	[3]
	Oleanolic and glycyrrhetinic acids derivatives	HeLa	↓cell viability (IC_{50} = 4.32 ± 0.89 μM)	—	[4]
	CDDO	HeLa	↑Gal4-PPARγ transactivation	—	[5]
	CDDO-Me	HeLa r0	↑TFAM, no effect on Lon expression, slightly ↑p53	—	[6]
	CDDO-Me	HeLa	↓IL6-induced and constitutive p-JAK1, direct interaction with JAK1, ↓IL6-induced STAT3, ↓IL6-induced p-STAT3, ↓IL6-induced STAT3 dimerization, ↓IL6-induced Cyclin D1	JAK1	[7]
	CDDO-Im	HeLa	siRNA (IRAK2) → ↓CDDO-Im-induced cell death	—	[8]
	CDDO-TFEA	HeLa	↓TNFα-induced NF-kB activity, slight effect on NF-kB basal activity	—	[9]
	CDDO-TFEA, CDDO-Me	HeLa	↓TNFα-induced NF-kB activity, ↓TNFα-induced p-IkBα	—	[10]
	Soloxolone methyl (SM)	KB-3-1, KB-8-5, HeLa	↓cell viability (IC_{50} = 0.3–1.3 μM), ↑nuclear condensation, ↑nuclear fragmentation, ↑PS[c] externalization, ↓$\Delta\psi_M$, caspase activation,	ROS (GSH co-treatment → ↓triterpenoid-induced cell death)	[11]
	Cyano-enone-bearing boswellic acid derivatives	HeLa	Cytotoxic (IC_{50} = 0.27 pM)	—	[12]
	Cyano-enone-bearing boswellic acid derivatives	HeLa	Cytotoxic (IC_{50} = 3-50 μM)	—	[13]
Ursane	Ursolic acid	C33A, HeLa, CaSki, SiHa	↓cell growth, ↑DNA fragmentation, ↑condensation and fragmentation of nuclei, ↑Sub-G1, ↑Fas, ↑cleaved caspases-8, -3, PARP, ↓HPV-18 E6/E7	—	[14]
	Ursolic acid	HeLa	↓cell viability (IC_{50} = 9.54 ± 0.98 μM), ↑PS externalization, ↑activity of caspases-9, -3, ↑cytochrome c release, ↑Bax, ↑Bak, ↓Bcl-2, ↓Bcl-xL, ↓p-p38, ↓p-ERK1/2, ↑DUSP 1, 2, 4, 5, 6, 7, 9, 10	ERK1/2 (U0126 pretreatment → ↓effect of ursolic acid on Bax/Bcl-2, cytochrome c release, cleavage of caspase-3)	[15]
	Ursolic acid + cisplatin	H8, HeLa, SiHa, C-33A, ME-180	↓cell growth, apoptosis, ↓Bcl-2, ↓NF-kB p65, ↓NF-kB p65 nuclear translocation, ↑Bax, ↑cleaved caspase-3 and PARP	—	[16]
	Ursolic acid into poly (DL-lactide-co0glycolide) nanoparticles	CaSki, HeLa, C4-1, SiHa	↓cell growth, ↓colony formation, ↓cell migration, ↑PS externalization, ↑cleaved caspases-8, -9, -3, ↑p53, ↑Bax/Bcl-2, ↑Fas, ↓cIAP-1; ↓SiHa, CaSki and HeLa xenograft growth, ↑p53, ↓Bcl-2, ↑survival rate	—	[17]

Type	Compound	Cell line	Effect	Upstream cell death-associated target	Ref.
Lupane	Betulinic acid	HeLa	Apoptosis, ↓PI3K p85, ↓PI3K p110a, ↓p-Akt, ↑ROS, G_0/G_1 arrest, ↑p27, ↑p21, ↑Bad, ↑caspase-9, ↓$\Delta\psi_M$	ROS (GSH pretreatment → ↓BA-induced apoptosis, ↓BA effect on PI3K (p85, p110a) and p-Akt)	[18]
	Betulinic acid	HeLa	↓cell viability (IC_{50} = 30.42 ± 2.39 µM), ↑apoptotic bodies, ↑PS externalization, 37 differentially expressed proteins involved in regulation of protein folding, glycolysis, translation and mRNA splicing (down-regulated proteins), ↑ROS, ↓14-3-3β, ↓14-3-3ε, ↑Bax/Bcl-2	—	[19]
	Betulinic acid	U14	↓tumor growth, ↓IL2, ↓TNFα, ↑CD4+ lymphocytes subsets, ↑apoptosis, ↓Bcl-2, ↓Ki-67	—	[20]
	Lupeol	HeLa, SiHa	↓cell growth, S arrest, ↓cyclin E, ↓cyclin A, ↑p21, ↑PS externalization, ↑Bax/Bcl-2, ↑cleaved PARP, ↓$\Delta\psi_{M,}$ ↑mitochondrial superoxides	ROS (NAC pretreatment → ↓lupeol-induced cell death)	[21]
	2α-bromo-dihydrobelulonic acid	HeLa	↓cell viability, ↑PS externalization, G_0/G_1 arrest, ↓cyclin D1, ↓PCNA, ↑21	—	[22]
	Mollic acid	CaSki	Apoptosis (nuclear shrinkage, condensation and fragmentation; ↑PS externalization, ↑apoptotic blebs), ↓$\Delta\psi_{M,}$ ↑Bax/Bcl-2	—	[23]
	Bitter melon triterpenoids	HeLa	↑p-AMPK, STO-609 (CAMKK inhibitor) pretreatment → complete blocking of triterpenoid-induced p-AMPK	—	[24]
	Lupane derivative 9	HeLa, CaSki	Cytotoxic (IC_{50} = 2.1 and 6.3 µM, respectively; $IC_{50}^{normal\ fibroblast}$ > 20 µM)	—	[25]
	Pyrazine derivatives of 23-hydroxybetulinic acid	HeLa	↓cell viability (IC_{50} = 5.13 µM)	—	[26]
Other	Pristimerin	HeLa, CaSki, SiHa	Cytotoxic (IC_{50} = 0.85–1.7 µM), ↑PS externalization, ↑cleaved caspases-9, -3, PARP-1, ↑cytochrome c release, ↑nuclear AIF, ↑Bax, siRNA (Bax) → ↓pristimerin-induced cell death, ↑p-JNK, ↑p-ERK, ↑p-p38, SP600125 pretreatment → ↓pristimerin-induced cell death, ↓effect of pristimerin on Bax, cytochrome c release, nuclear AIF, cleavage of caspases-9,-3; ↑ROS	ROS (NAC pretreatment → ↓effect of pristimerin on Bax, cytochrome c release, cleavage of caspases-9,-3, PARP-1, p-JNK)<br>PARP (DIQ or siRNA (PARP) → ↓pristimerin-induced cell death)	[27]
	Ganoderic acid derivatives	HeLa	↓cell viability (IC_{50} = 4.1 µM), ↓DNA synthesis, ↑nuclear condensation, ↑subG1, ↑PS externalization, ↓$\Delta\psi_{M,}$ ↑activity of caspases-9, -3	—	[28]
	3-acetoxylanosta-8,24-dien-21-oic acid (FPOA)	HeLa	↓cell viability (IC_{50} = 25.28 µg/ml), ↑apoptotic bodies, ↑PS externalization, ↑ROS, ↑cleaved caspases-9, -3, PARP, ↑Bax/Bcl-2	—	[29]
	Inotodiol	HeLa	↓cell viability (IC_{50} = 116.5 µg/ml), ↑cell shrinkage, ↑cell rounding, ↑subG1, ↓cyclin E, ↑p27, ↑Bax/Bcl-2	—	[30]

(a) Published reports were searched by using search query with the keywords “triterpenoid AND cervical”, “CDDO AND cervical”, “triterpenoid AND HeLa”, “triterpenoid AND SiHa”, “CDDO AND HeLa” in Google Scholar and SCOPUS search engines (search period was from 2000 to December 2018), followed by the articles described pentacyclic triterpenoids were selected (b)ΔψM is defined as mitochondrial membrane potential; (c)PS is defined as posphatidylserine;
